# Supplementary material for: Interfacial engineering of Bi2S3/Ti3C2Tx MXene based on work function for rapid photo-excited bacteria-killing
Source: Nat Commun. 2021 Feb 22;12:1224. doi: 10.1038/s41467-021-21435-6 (PMC7900204; doi:10.1038/s41467-021-21435-6)
Supplement: Supplementary file 1 — Supplementary Information [file 41467_2021_21435_MOESM1_ESM.pdf]

Supplementary Information

Interfacial engineering of $Bi_2S_3/Ti_3C_2T_x$ MXene based on work function for rapid photo-excited bacteria-killing

Li *et al.*

Supplementary Materials:

Supplementary Figs. 1-33

Supplementary Table 1-2

Supplementary Methods

Supplementary References 1-23

Supplementary Figures

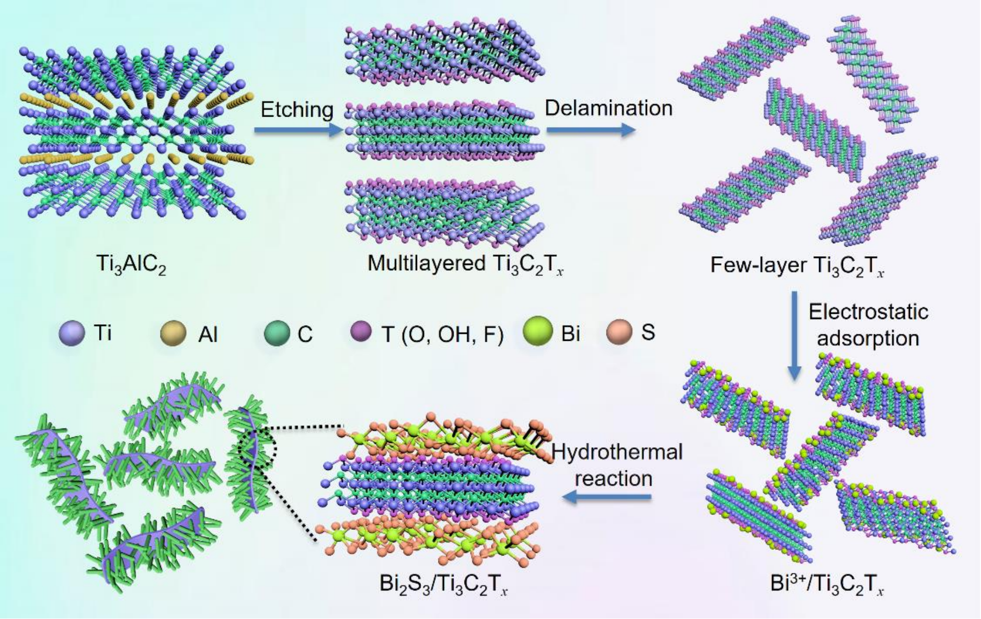

Supplementary Fig. 1 | The schematic illustration of $Bi_2S_3/Ti_3C_2T_x$ Schottky junction.

As shown in Supplementary Fig. 1, the bulk Ti_3AlC_2 was etched by 40% HF firstly to obtain the multilayer $Ti_3C_2T_x$. After intercalation and ultrasonic exfoliation, few-layer nanosheets can be prepared. During this process, the surface of $Ti_3C_2T_x$ nanosheets was endowed with some functional groups (e.g., -F, -OH, and -O), resulting in negatively charged and hydrophilic performance, which is beneficial to the subsequent metal cation adsorption of Bi^{3+}. So Bi^{3+} ions were electrostatically adsorbed onto the negatively charged surface of the $Ti_3C_2T_x$ nanosheets to ensure in situ growth of Bi_2S_3 nanorods during hydrothermal treatment. During hydrothermal treatment, the nucleation and growth of Bi_2S_3 nanorods are based on Bi^{3+} and S^{2-} ions released slowly from the precursor solution. Thioacetamide (TAA) is used as both sulfur source and ligand, which can form complexes $[Bi(TAA)_n]^{3+}$ with Bi^{3+}. During hydrothermal process, the following reactions may occur in the solution.

$H_3CCSNH_2 + H_2O \rightarrow CH_3CONH_2 + H_2S$;

$H_2S \rightarrow HS^- + H^+$;

$HS^- \rightarrow S^{2-} + H^+$;

$3\ S^{2-} + 2\ [Bi(TAA)_n]^{3+} \rightarrow Bi_2S_3 + n\ TAA$.

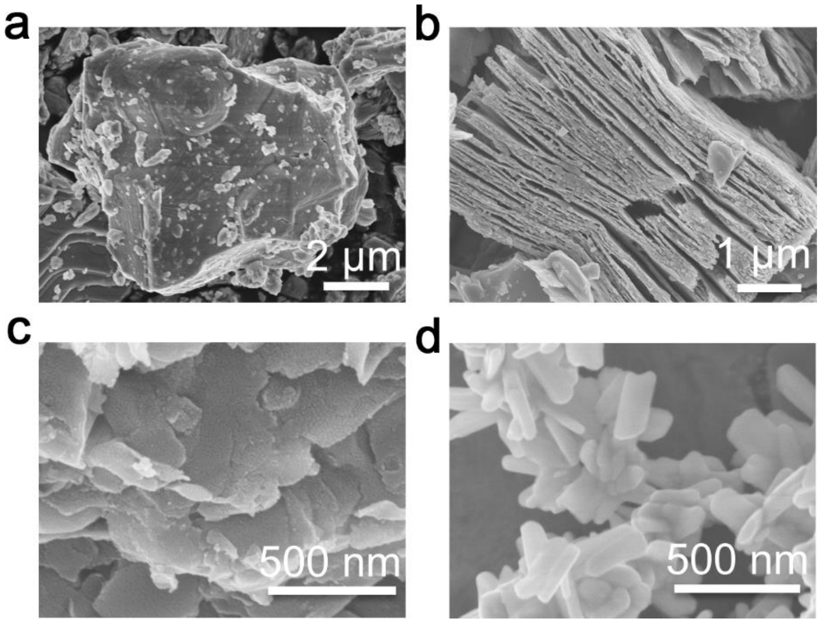

Supplementary Fig. 2 | SEM images of $Ti_3C_2T_x$ and Bi_2S_3. a, b, Ti_3AlC_2 before (**a**) and after (**b**) HF etching. **c,** $Ti_3C_2T_x$ MXene sheet after exfoliation. **d,** Bi_2S_3.

Compared to bulk Ti_3AlC_2 (Supplementary Fig. 2a), the multilayer $Ti_3C_2T_x$ exhibited accordion-shape structure (Supplementary Fig. 2b). Few-layer nanosheets can be seen in Supplementary Fig. 2c after intercalation and ultrasonic exfoliation. Pristine Bi_2S_3 showed nanorods structure (Supplementary Fig. 2d).

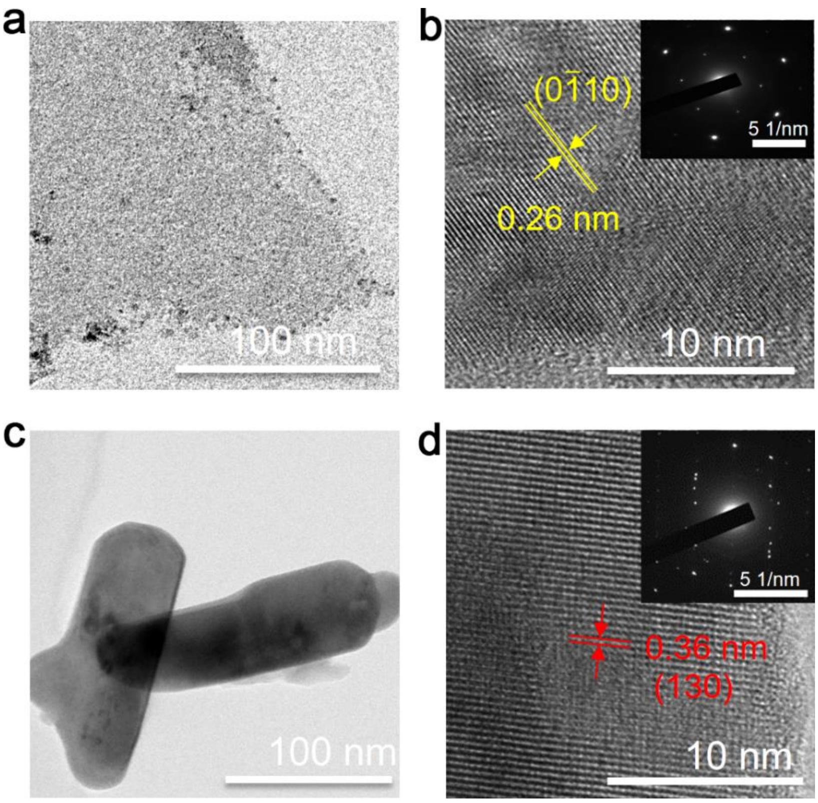

Supplementary Fig. 3 | TEM images of $Ti_3C_2T_x$ and Bi_2S_3. a, b, TEM (**a**) and HRTEM (**b**) patterns of exfoliated $Ti_3C_2T_x$ MXene (Inset presents SAED pattern.). **c, d,** TEM (**c**) and HRTEM (**d**) of Bi_2S_3.

The TEM of $Ti_3C_2T_x$ exhibited that $Ti_3C_2T_x$ was a very thin and transparent nanosheet with an inner-plane spacing of 0.26 nm, corresponding to the (0$\bar{1}$10) facet of crystallized $Ti_3C_2T_x$ with hexagonal structure[1] (Supplementary Fig. 3a, b). TEM image and HRTEM of Bi_2S_3 disclosed the nanorod-like structure of Bi_2S_3, which exhibited a lattice fringes of the (130) crystal plane with a d-spacing of 0.36 nm, belonging to the orthorhombic Bi_2S_3[2] (Supplementary Fig. 3c, d).

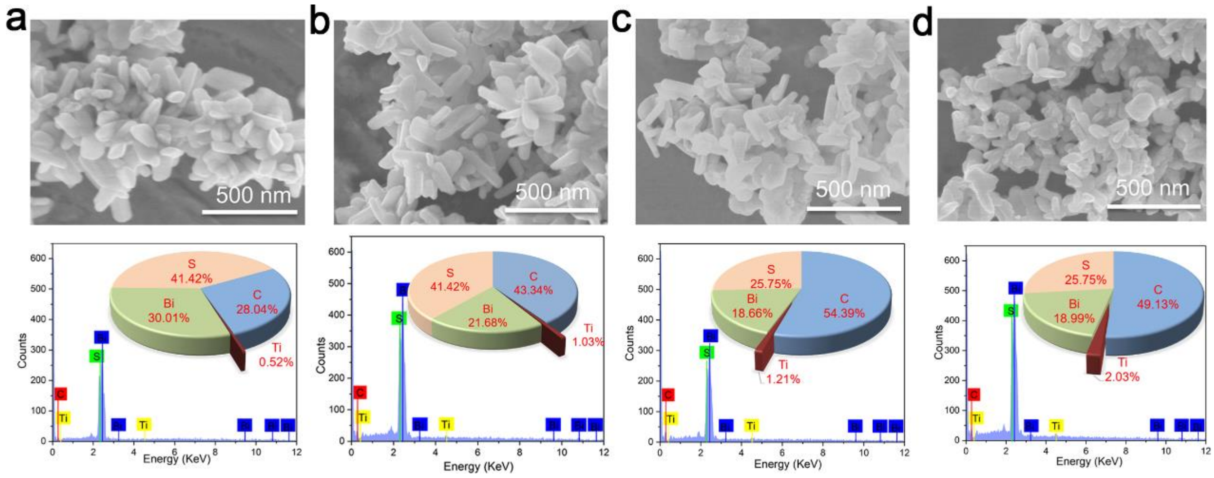

Supplementary Fig. 4 | SEM images and corresponding EDS analysis. a-d, SEM images and EDS results of $Bi_2S_3/Ti_3C_2T_x$-2 (**a**), $Bi_2S_3/Ti_3C_2T_x$-5 (**b**), $Bi_2S_3/Ti_3C_2T_x$-7 (**c**), and $Bi_2S_3/Ti_3C_2T_x$-10 (**d**). No obvious differences can be observed for Bi_2S_3 and $Bi_2S_3/Ti_3C_2T_x$-x in SEM images. It can be inferred that the clubbed structure of Bi_2S_3 may completely covered the ultrathin $Ti_3C_2T_x$ nanosheets. And the molar ratio of Ti and Bi gradually increased as the increase of $Ti_3C_2T_x$. Source data are provided as a Source Data file.

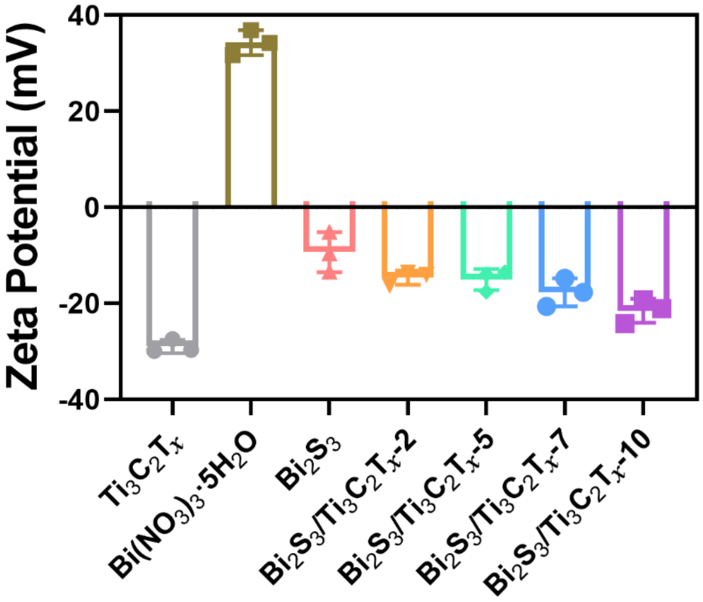

Supplementary Fig. 5 | Zeta potential. Zeta potential of the $Ti_3C_2T_x$ suspension, $Bi(NO)_3{\cdot}5H_2O$, $Bi_2S_3/Ti_3C_2T_x$-2, $Bi_2S_3/Ti_3C_2T_x$-5, $Bi_2S_3/Ti_3C_2T_x$-7, and $Bi_2S_3/Ti_3C_2T_x$-10. Data are shown as mean ± standard deviations; n = 3 independent samples. Source data are provided as a Source Data file.

The measured Zeta potential shown in Supplementary Fig. 5 verified this electrostatic interaction between two components during the preparation, *i.e.*, Bi_2S_3 grew in situ on the surface of $Ti_3C_2T_x$ through the two procedures. Firstly, electrical interaction can occur between Bi^{3+} and the functional groups on the surface of $Ti_3C_2T_x$ as $Ti_3C_2T_x$ nanosheets were negatively charged (−37.72 ± 1.24 mV). And then, the nanorods uniformly formed during hydrothermal process, thus avoiding the aggregation and the restacking between $Ti_3C_2T_x$ nanosheets.

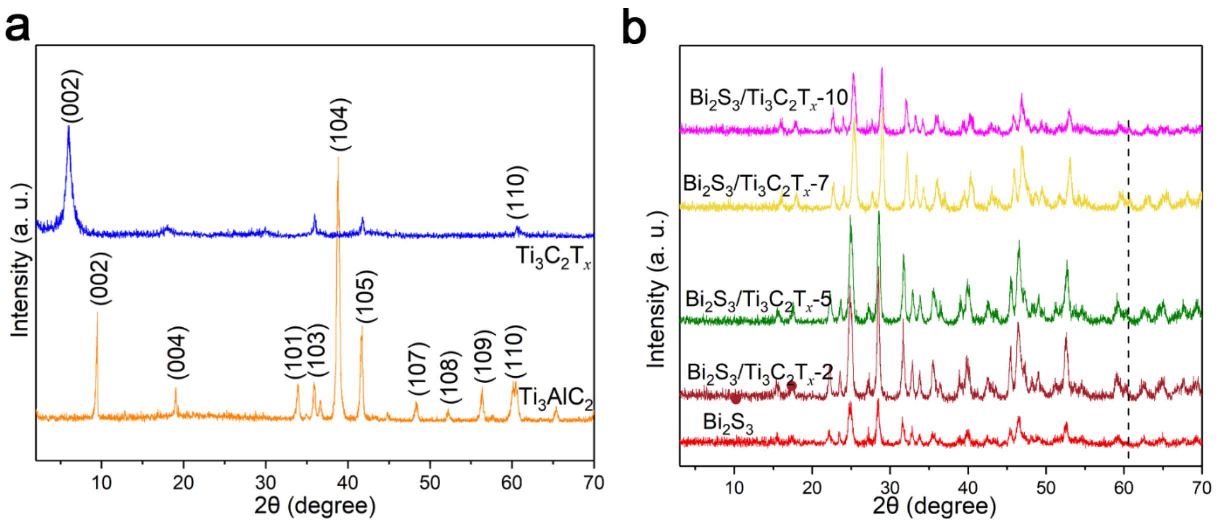

Supplementary Fig. 6 | XRD patterns. a, XRD patterns of Ti_3AlC_2 and $Ti_3C_2T_x$. **b,** XRD patterns of Bi_2S_3, $Bi_2S_3/Ti_3C_2T_x$-2, $Bi_2S_3/Ti_3C_2T_x$-5, $Bi_2S_3/Ti_3C_2T_x$-7, and $Bi_2S_3/Ti_3C_2T_x$-10. Source data are provided as a Source Data file.

X-ray diffraction (XRD) spectra shown in Supplementary Fig. 6 disclosed that the prominent diffraction peak (104) of $Ti_3C_2T_x$ centered at 39° disappeared compared with the Ti_3AlC_2, suggesting that the Al element was successfully removed after HF etching[3]. So the bulk Ti_3AlC_2 was almost completely converted into $Ti_3C_2T_x$ MXene. The diffraction peak of (002) peak broadened and shifted from 9.5° toward a lower angle of 6.07°, corresponding to the increased interlayer of d-spacing from 9.2 to 14.54 Å. The XRD patterns of $Bi_2S_3/Ti_3C_2T_x$ with different contents of $Ti_3C_2T_x$ (Supplementary Fig. 6b) showed that these peaks contain all the peaks of pristine Bi_2S_3[4,5]. In addition, the tiny peaks at about 61° for 2%, 5%, 7% and 10% $Bi_2S_3/Ti_3C_2T_x$ are ascribed to the (110) plane of $Ti_3C_2T_x$ MXenes[6, 7]. In comparison with Bi_2S_3, these peaks intensity of Bi_2S_3 in $Bi_2S_3/Ti_3C_2T_x$-x increased, suggesting the better crystallization, which may be ascribed to the fact that the Bi^{3+} ions adsorbed on the $Ti_3C_2T_x$ nanosheets was used as the nucleation sites of

Bi_2S_3, further accelerating the growth of Bi_2S_3 nanorods. Moreover, the peak intensity weakened as the content of $Ti_3C_2T_x$ increases, which could be attributed to the decreased grain size[8].

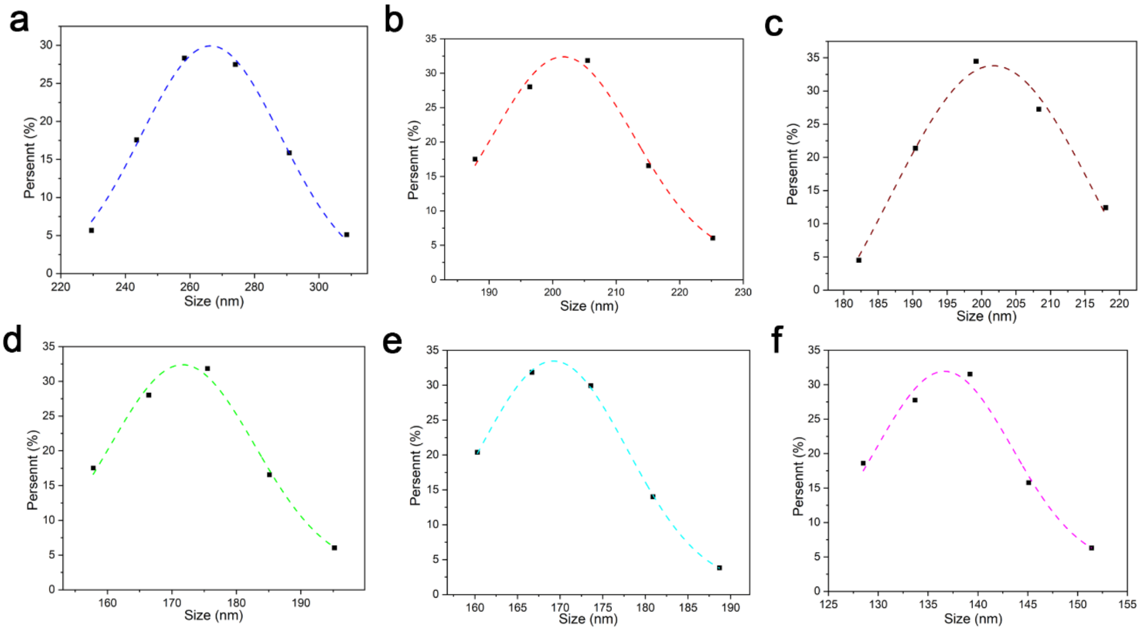

Supplementary Fig. 7 | Hydrodynamic sizes. a-f, Hydrodynamic sizes of $Ti_3C_2T_x$ (**a**), Bi_2S_3 (**b**), $Bi_2S_3/Ti_3C_2T_x$-2 (**c**), $Bi_2S_3/Ti_3C_2T_x$-5 (**d**), $Bi_2S_3/Ti_3C_2T_x$-7 (**e**), and $Bi_2S_3/Ti_3C_2T_x$-10 (**f**). (n = 1) The diameter of $Bi_2S_3/Ti_3C_2T_x$ gradually decreased with the increase of $Ti_3C_2T_x$ content. Source data are provided as a Source Data file.

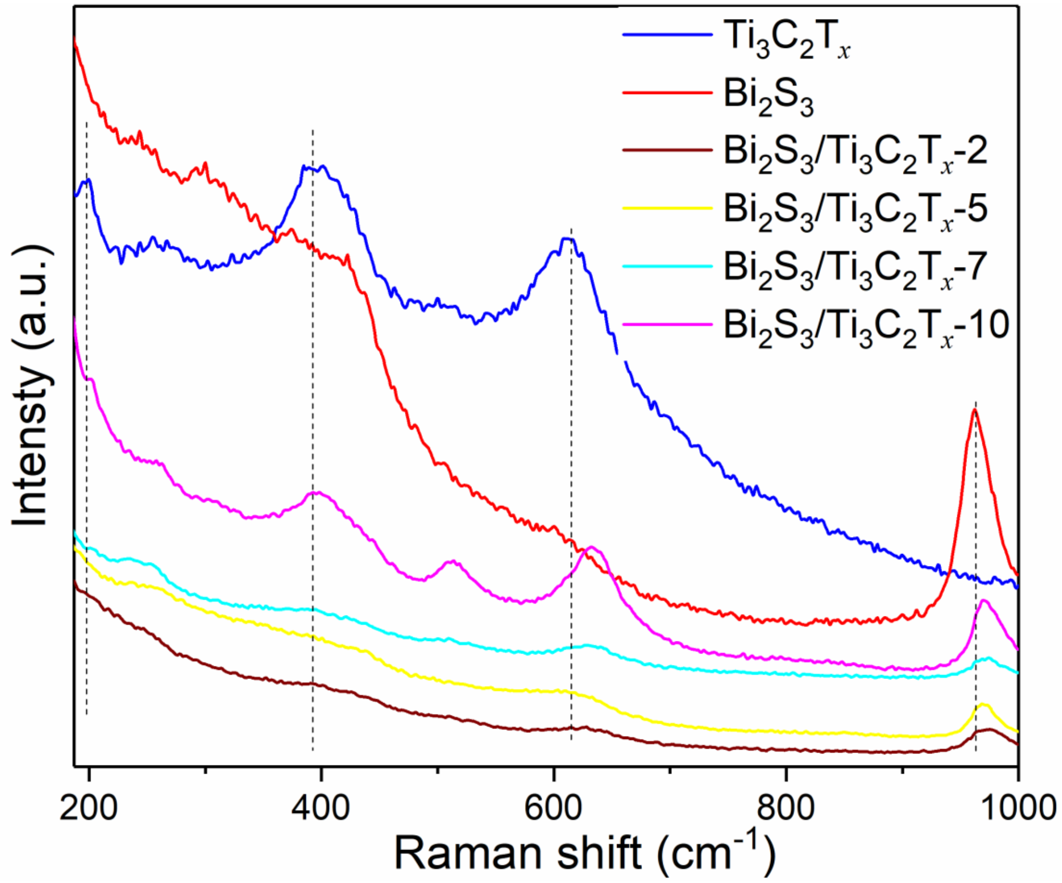

Supplementary Fig. 8 | Raman spectra. Raman spectra of $Ti_3C_2T_x$, Bi_2S_3, $Bi_2S_3/Ti_3C_2T_x$-2, $Bi_2S_3/Ti_3C_2T_x$-5, $Bi_2S_3/Ti_3C_2T_x$-7, and $Bi_2S_3/Ti_3C_2T_x$-10. Source data are provided as a Source Data file.

The Raman spectra of samples were provided in Supplementary Fig. 8, Raman bands located at 198 cm^{-1}, 393 cm^{-1}, and 615 cm^{-1} can be observed, matching with the Raman feature of $Ti_3C_2T_x$[9]. And the band at 965 cm^{-1} belongs to the Raman feature of Bi_2S_3[10]. These Raman peaks of $Ti_3C_2T_x$ and Bi_2S_3 can be observed in $Bi_2S_3/Ti_3C_2T_x$-x, further verifying the presence of $Ti_3C_2T_x$ in the $Bi_2S_3/Ti_3C_2T_x$-x.

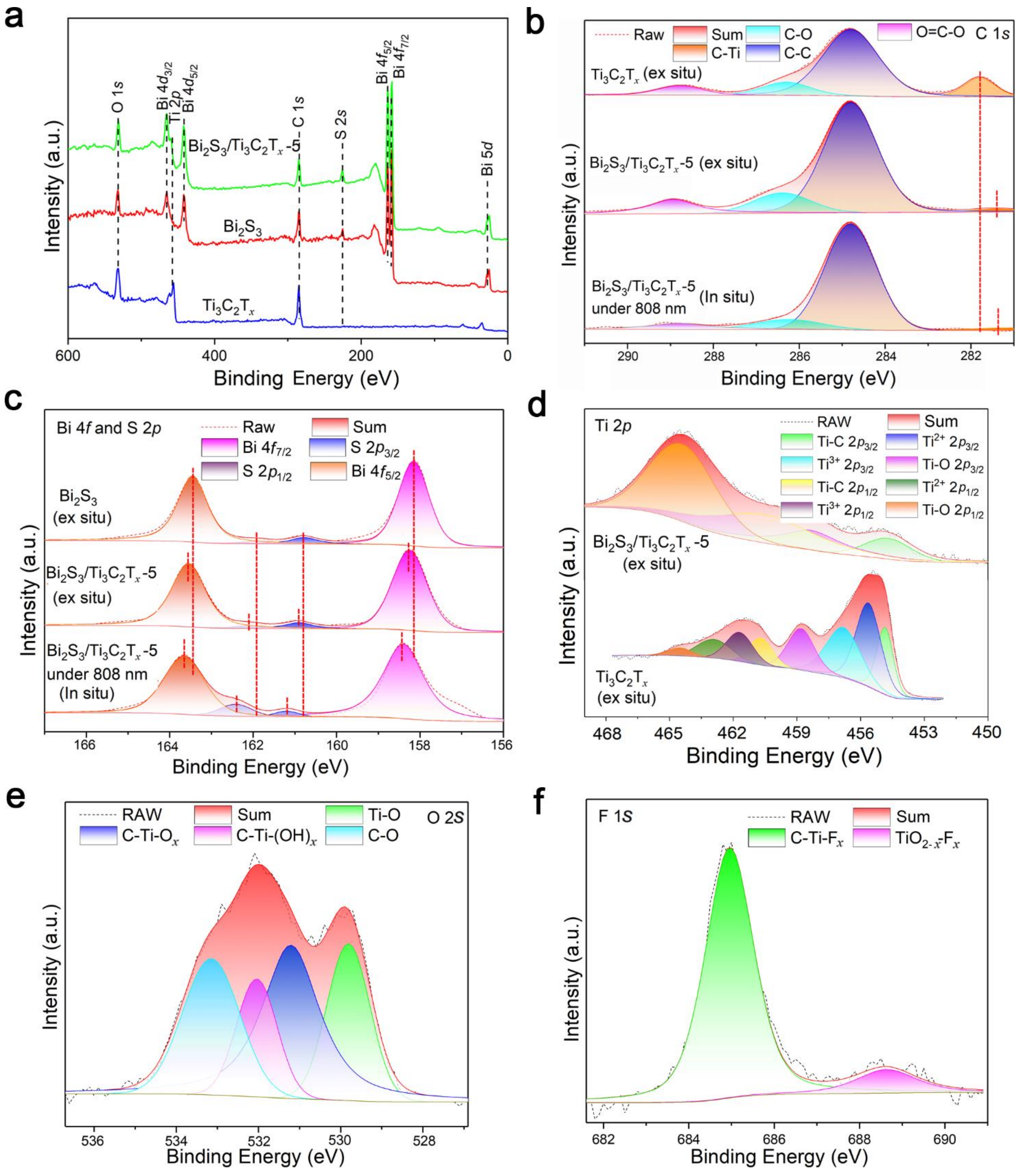

Supplementary Fig. 9 | In situ and ex situ XPS measurements of as-synthesized samples. a, XPS survey scan. **b-d,** High-resolution of C 1*s* (**b**), Bi 4*f* and S 2*p* (**c**) and Ti 2*p* (**d**). **e, f,** High-resolution of O 2*s* (**e**) and F 1*s* (**f**) for $Ti_3C_2T_x$. Source data are provided as a Source Data file.

The signal of Ti, C, Bi, and S detected from $Bi_2S_3/Ti_3C_2T_x$-5 by XPS survey scan (Supplementary Fig. 9a) suggested the successful combination between $Ti_3C_2T_x$ and Bi_2S_3. The ex situ C 1*s* exhibited four peaks, corresponding to the C-C, C-O, Ti-C, and O=C-O bonds, respectively[11]. The O=C-O peak may be from contamination for samples exposed to air[12, 13]. Because the content of $Ti_3C_2T_x$ is very low, the peak of Ti-C is relatively low. The Bi 4*f* and S 2*p* reveal four peaks assigned to Bi $4f_{7/2}$, S $2p_{3/2}$, S $2p_{1/2}$ and Bi $4f_{7/2}$[14]. The Ti 2*p* spectrum of $Ti_3C_2T_x$ was fitted with four Ti species, assigned to Ti-O, Ti^{2+}, Ti^{3+}, and Ti-C. As a contrast, $Bi_2S_3/Ti_3C_2T_x$-5 only showed two Ti species (Ti-O and Ti-C). Obviously, the binding energies of Ti-C peaks for C 1*s* and Ti 2*p* shifted to low binding energy compared to those of $Ti_3C_2T_x$, whereas the Bi 4*f* and S 2*p* of $Bi_2S_3/Ti_3C_2T_x$-5 shifted to higher binding energy compared to those of Bi_2S_3. All these results confirmed the strong interface interaction at the interface between $Ti_3C_2T_x$ and Bi_2S_3, resulting in the decrease of the local electron density of Bi and S centers and the increase of the one of Ti centers and the followed electrons transfer from Bi_2S_3 to $Ti_3C_2T_x$ when $Ti_3C_2T_x$ and Bi_2S_3 contact tightly. Consequently, Schottky junction and built-in electric field formed at the interface between $Ti_3C_2T_x$ and Bi_2S_3[15]. In addition, the charge transfer under 808 nm light irradiation was evidenced by the in situ XPS. The binding energy of Ti-C for $Bi_2S_3/Ti_3C_2T_x$-5 under 808 nm light irradiation shifted negatively with reference to that in ex situ spectra. Conversely, the peaks of Bi 4*f* and S 2*p* shifted positively. This shift demonstrates that the photogenerated electrons in Bi_2S_3 can transfer to $Ti_3C_2T_x$ under 808 nm light irradiation. The high-resolution spectra of O 1*s* and F 1*s* obtained from $Ti_3C_2T_x$ (Supplementary Fig. 9e, f) disclosed that the ratio between the F: O: OH functional group was 0.97: 1.46: 0.58, indicating the random distribution of F, OH, and O groups on the surface of $Ti_3C_2T_x$.

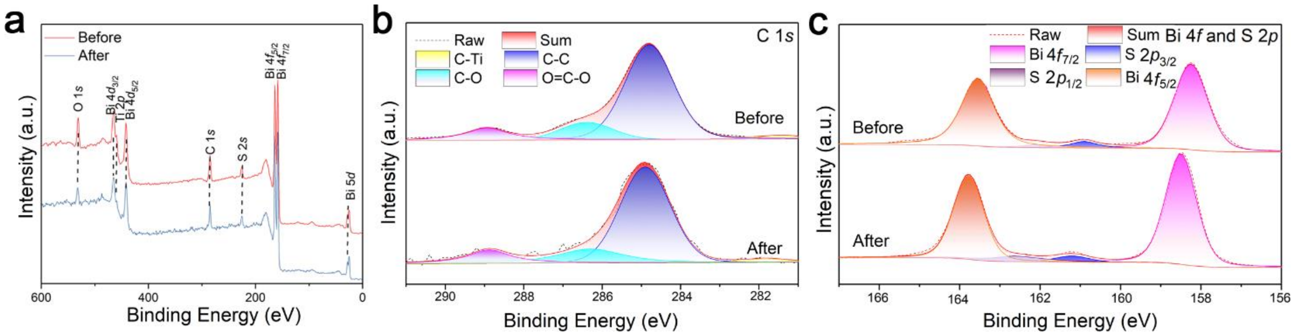

Supplementary Fig. 10 | XPS measurements of $Bi_2S_3/Ti_3C_2T_x$-5 before and after a month. a, XPS survey scan. **b, c,** High-resolution of C 1*s* (**b**) and Bi 4*f* and S 2*p* (**c**). Source data are provided as a Source Data file.

In order to systematically evaluate the stability of $Bi_2S_3/Ti_3C_2T_x$, XPS was measured before and after one month. There was no change for the signal of Ti, C, Bi, and S from XPS survey scan (Supplementary Fig. 10a). In addition, both of them exhibited C-C, C-O, Ti-C and O=C-O bonds from the high-resolution spectra of C 1*s* (Supplementary Fig. 10b). Similar, the high-resolution spectra of Bi 4*f* and S 2*p* reveal four peaks assigned to Bi $4f_{7/2}$, S $2p_{3/2}$, S $2p_{1/2}$ and Bi $4f_{7/2}$ (Supplementary Fig. 10c).

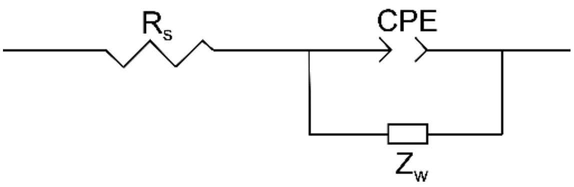

Supplementary Fig. 11 | Electronic equivalent circuit used in the fitting procedure of EIS data.

An equivalent circuit model is shown in Supplementary Fig. 11, which was composed of solution resistance (R_s), double-layer capacitance (CPE) and Warburg impedance (Z_w). The result of fitting was listed in Supplementary Table 1. In the low-frequency range, all the different electrodes exhibited a linear line, suggested the ion diffusion. The smallest Z_w value of $Bi_2S_3/Ti_3C_2T_x$-5 reflected better conductivity to promote electron transfer of electrode surface.

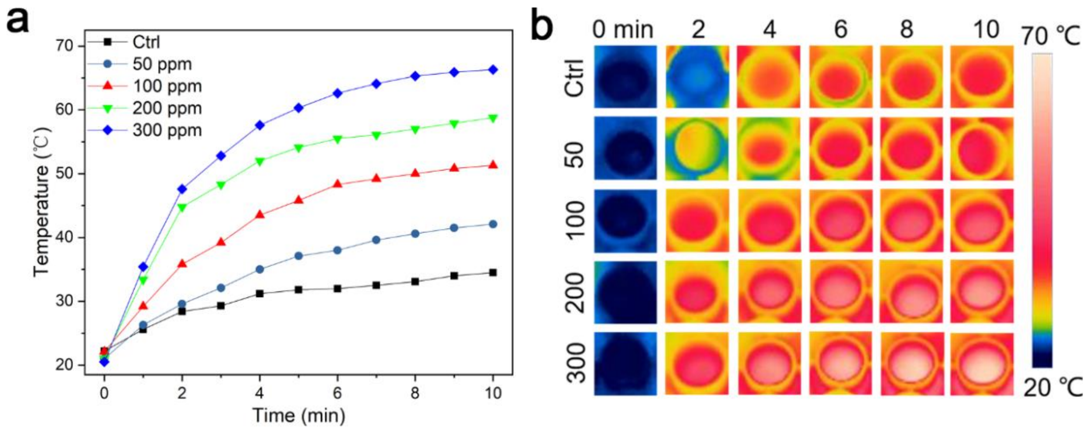

Supplementary Fig. 12 | Photothermal measurement and corresponding real-time infrared thermal images. a, Photothermal measurement of the different concentrations of $Bi_2S_3/Ti_3C_2T_x$-5 in the PBS solution under 808 nm NIR (0.7 W cm^{-2}) for 10 min. **b,** The corresponding real-time infrared thermal images for different concentrations of $Bi_2S_3/Ti_3C_2T_x$-5 solution. Source data are provided as a Source Data file.

As shown in Supplementary Fig. 12, the temperature of $Bi_2S_3/Ti_3C_2T_x$-5 was increased with the increase of concentrations under 808 nm NIR light irradiation. Among these concentrations of $Bi_2S_3/Ti_3C_2T_x$-5, the 300 ppm group exhibited the highest temperature of up to 65 °C under 10 min irradiation while the temperature of 200 ppm group rose to 58.5 °C under the same condition. The former may cause severe burns of skin and deep tissues easily in a short time[16]. So the latter was chosen as the ultimate concentration.

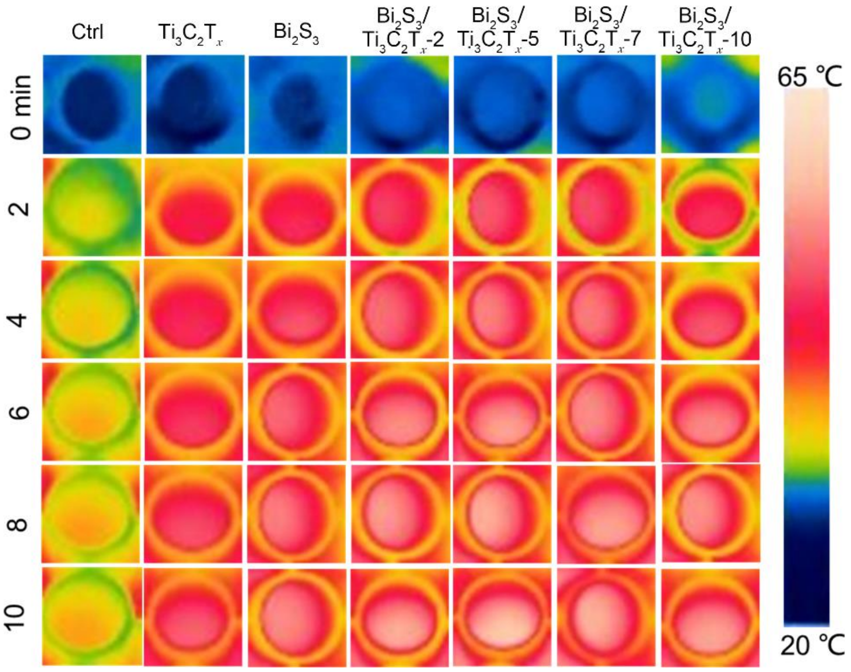

Supplementary Fig. 13 | The real-time infrared thermal images. The real-time infrared thermal images of $Ti_3C_2T_x$, Bi_2S_3, $Bi_2S_3/Ti_3C_2T_x$-2, $Bi_2S_3/Ti_3C_2T_x$-5, $Bi_2S_3/Ti_3C_2T_x$-7, and $Bi_2S_3/Ti_3C_2T_x$-10 at concentration of 200 ppm under 808 nm near infrared light irradiation.

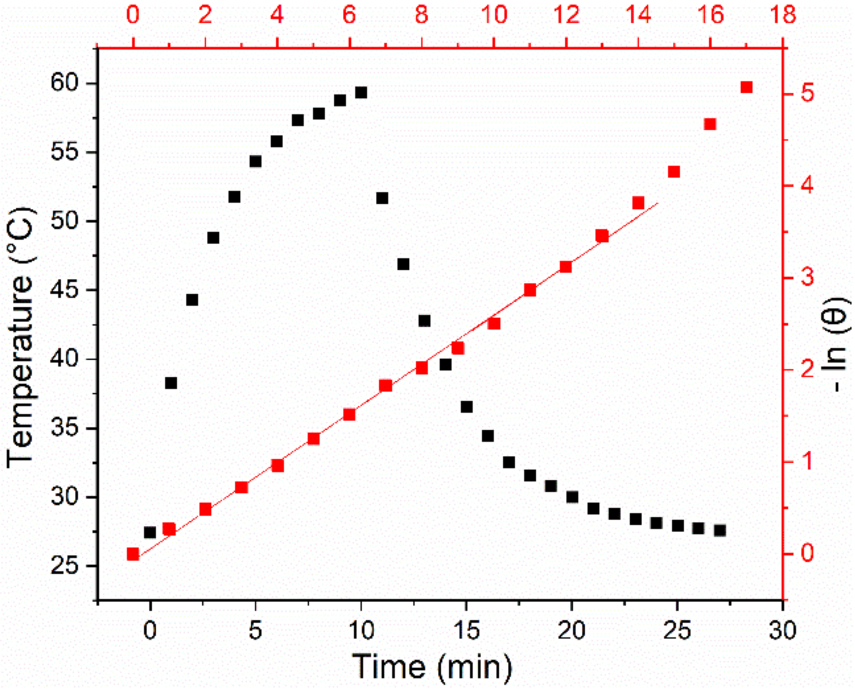

Supplementary Fig. 14 | Calculation of the photothermal-conversion efficiency (η) of $Bi_2S_3/Ti_3C_2T_x$-5 under 808 nm NIR irradiation. Source data are provided as a Source Data file.

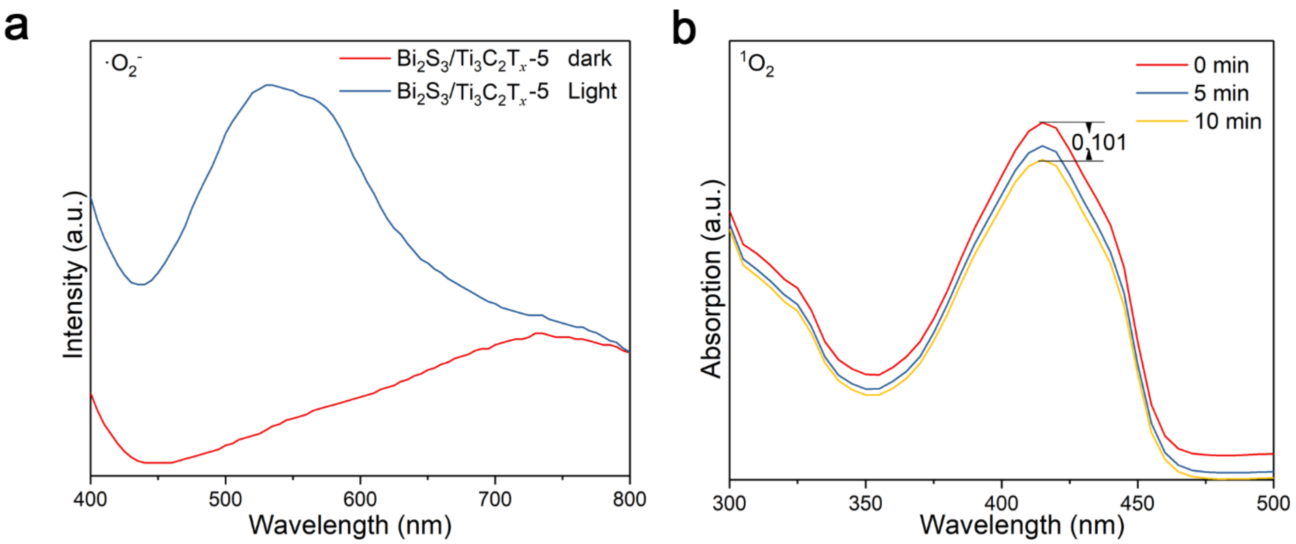

Supplementary Fig. 15 | $\cdot O_2^-$ and $^1O_2$ characterization. a, The absorption spectra of MF in the dark or 808 nm light. **b,** The detection of $^1O_2$ using DPBF as detector under light irradiation. Source data are provided as a Source Data file.

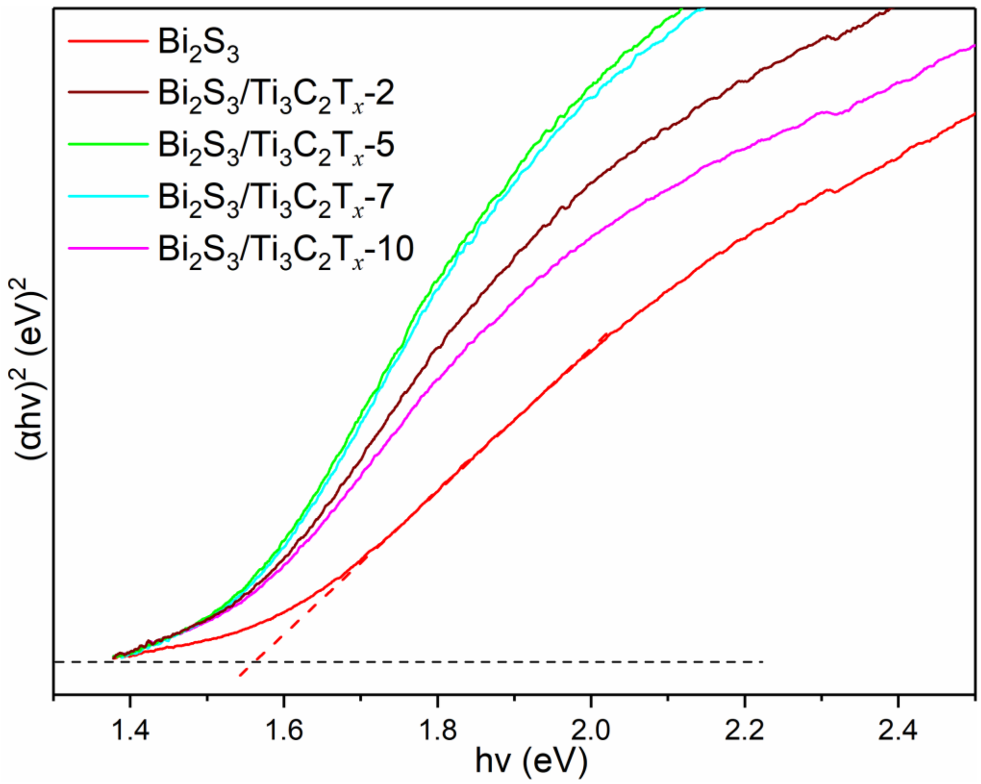

Supplementary Fig. 16 | Energy gap calculated from UV-vis-NIR diffuse reflectance spectra. Source data are provided as a Source Data file.

The energy gap (E_g) can be obtained by the equation (6).

$$(\alpha h\nu)^n = B(h\nu - E_g) \qquad (6)$$

W here α is absorption coefficient, $h\nu$ denotes the energy of incident photons, n is a an index associated with the nature of the transition to n = 2 for direct semiconductor and n = 1/2 for indirect semiconductor. B is a direct transition constant. So the E_g of Bi_2S_3 can be estimated by extrapolating the linear part of the spectrum of the plotting $(\alpha h\nu)^2$ versus $h\nu$. From Supplementary Fig. 16, the E_g of Bi_2S_3 was 1.56 eV, similar to previously reported value[17], which was larger than those of all $Bi_2S_3/Ti_3C_2T_x$ junctions, suggesting the low excitation energy of these Schottky junctions.

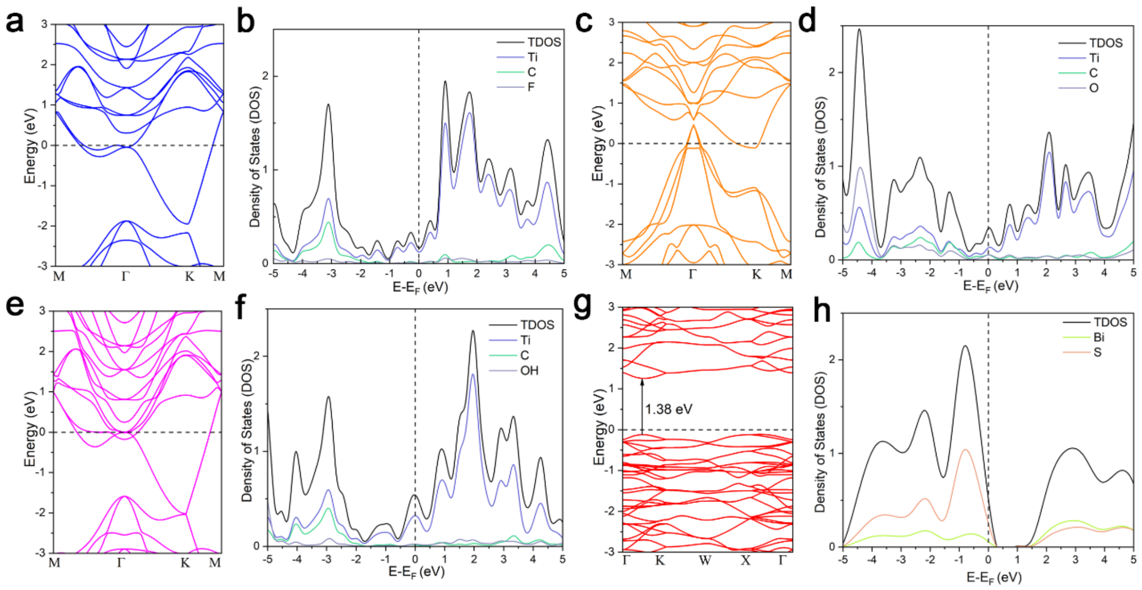

Supplementary Fig. 17 | Electronic band gap structure and corresponding DOS. a, b, Electronic band gap structure (**a**) and corresponding DOS (**b**) of $Ti_3C_2F_2$. **c, d,** Electronic band gap structure (**c**) and corresponding DOS (**d**) of $Ti_3C_2O_2$. **e, f,** Electronic band gap structure (**e**) and corresponding DOS (**f**) of $Ti_3C_2(OH)_2$. **g, h,** Electronic band gap structure (**g**) and corresponding DOS (**h**) of Bi_2S_3. Source data are provided as a Source Data file.

The electronic band gap and corresponding density of states (DOS) for F-terminated, O-terminated, and OH-terminated $Ti_3C_2T_x$ and Bi_2S_3 were calculated in Supplementary Fig. 17. The electronic band gap structure graphs exhibit spin-up and spin-down bands across the Fermi level, and the successive electronic states crossing the Fermi level for the corresponding DOS graphs are clearly observed, indicating typical metallic characteristics and excellent electrical conductivities of the F-terminated (Supplement Fig. 17a and 17b), O-terminated (Supplement Fig. 17c and 17d), and OH-terminated (Supplement Fig. 17e and 17f) $Ti_3C_2T_x$. This metallic property originated from the unsaturated Ti dangling bonds, which endowed $Ti_3C_2T_x$ with outstanding conductivity to transfer charges even after the inclusion of different functional groups. The electronic band gap structure

and corresponding DOS of Bi_2S_3 are plotted in Supplementary Fig. 17g and 17h. The band gap of this material was 1.38 eV. The DOS of the valence band was mainly contributed by the S atoms, while the DOS of the conduction band was provided by the hybridization of the S and Bi atoms. This band gap value was smaller than the experimental value in this work due to the insufficient exchange correlation of the generalized gradient approximation method[18].

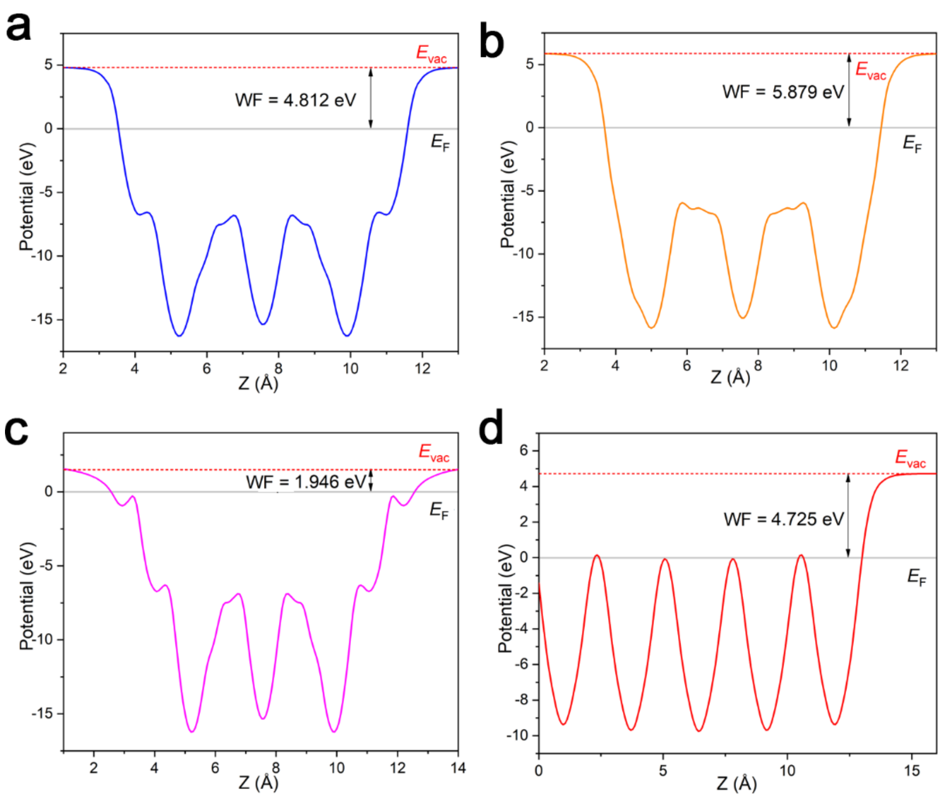

Supplementary Fig. 18 | Work function. a-d, The work function of $Ti_3C_2F_2$ (**a**), $Ti_3C_2O_2$ (**b**), $Ti_3C_2(OH)_2$ (**c**), and Bi_2S_3 (**d**). The red dashed lines denote vacuum level (E_{vac}), the gray line represent Femi level (E_F). Source data are provided as a Source Data file.

According to the average potential profile result, the WF values of $Ti_3C_2F_2$, $Ti_3C_2O_2$, $Ti_3C_2(OH)_2$, and Bi_2S_3 were calculated to be 4.812 eV, 5.879 eV, 1.946 eV, and 4.725 eV, respectively. The highest WF value (for $Ti_3C_2O_2$) among the $Ti_3C_2T_x$ samples suggested the strongest capacity to accept the photo-excited electrons from the semiconductor catalyst[19]. The calculated theoretical WF value of $Ti_3C_2T_x$ was 4.79 eV according to the XPS results, and it showed good agreement with the UPS results. Obviously, this theoretical WF value of $Ti_3C_2T_x$ was also higher than that of Bi_2S_3 (4.725 eV), pointing to the formation of a contact potential difference at the interface when Bi_2S_3 contacted $Ti_3C_2T_x$ tightly, and accelerating the transfer of charges across the interface.

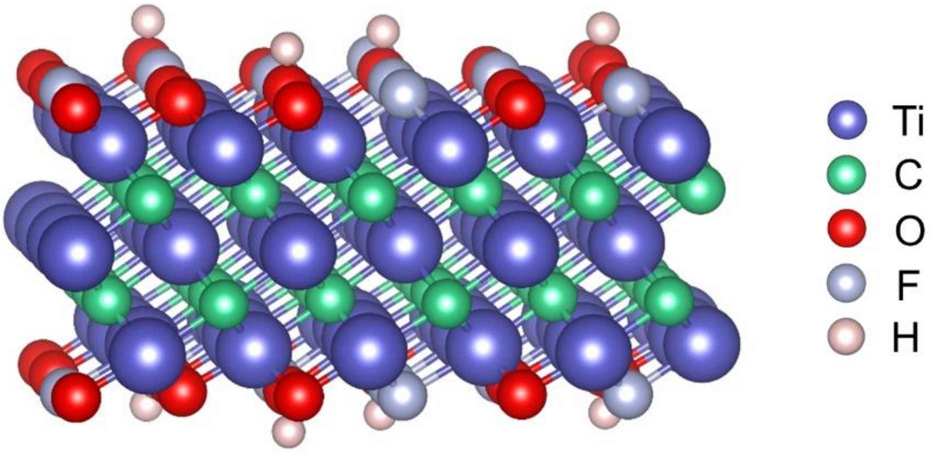

Supplementary Fig. 19 | Structure of the MXene slab. Structure of the MXene slab with mixed F, O and OH functional groups randomly distributed with relative amount 32: 49: 19 in agreement with XPS results. The DFT simulations of $Ti_3C_2T_x$ were carried out by a slab configuration with 1.5 nm of vacuum. It assumed that the surface of $Ti_3C_2T_x$ MXene was completely functionalized and symmetrical for top and bottom surfaces.

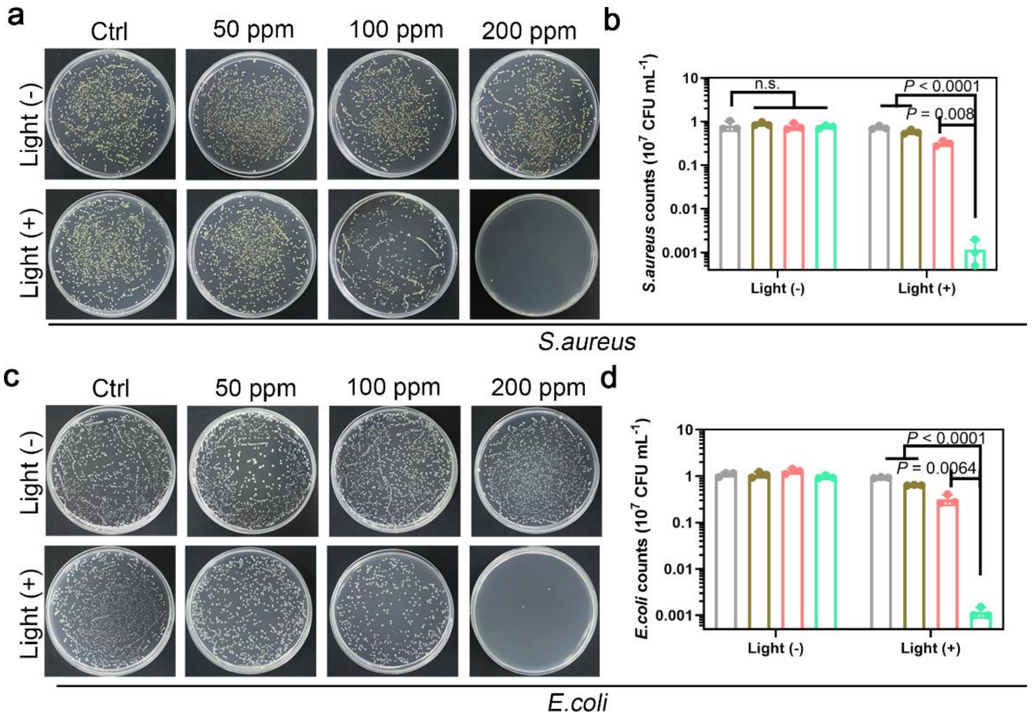

Supplementary Fig. 20 | Photographs of bacterial colonies and corresponding strain counts. a, b, Spread plate results (**a**) and corresponding strain counts (**b**) of *S. aureus*. **c, d,** Spread plate results (**c**) and corresponding strain counts (**d**) of *E. coli* treated with different concentration of $Bi_2S_3/Ti_3C_2T_x$-5 under 808 nm irradiation for 10 min after 24 h co-culturing. Data are presented as mean ± standard deviations from a representative experiment (n = 3 independent samples). P values were analysed by one-way ANOVA with Tukey's multiple comparisons post hoc test. Grey circles indicate the group of Ctrl, brown circles indicate the group of 50 ppm, pink circles indicate the group of 100 ppm, and green circles indicate the group of 200 ppm. Source data are provided as a Source Data file.

Supplementary Fig. 20 exhibited the antibacterial performance with different concentration of $Bi_2S_3/Ti_3C_2T_x$-5. The bacteria could not be killed effectively when the concentration of $Bi_2S_3/Ti_3C_2T_x$-5 with 50 ppm or 100 ppm for both *S. aureus* and *E. coli*. And $Bi_2S_3/Ti_3C_2T_x$-5 with

the concentration of 200 ppm exhibited best antibacterial activity. Thus, 200 ppm was chosen as the ultimate concentration for different samples.

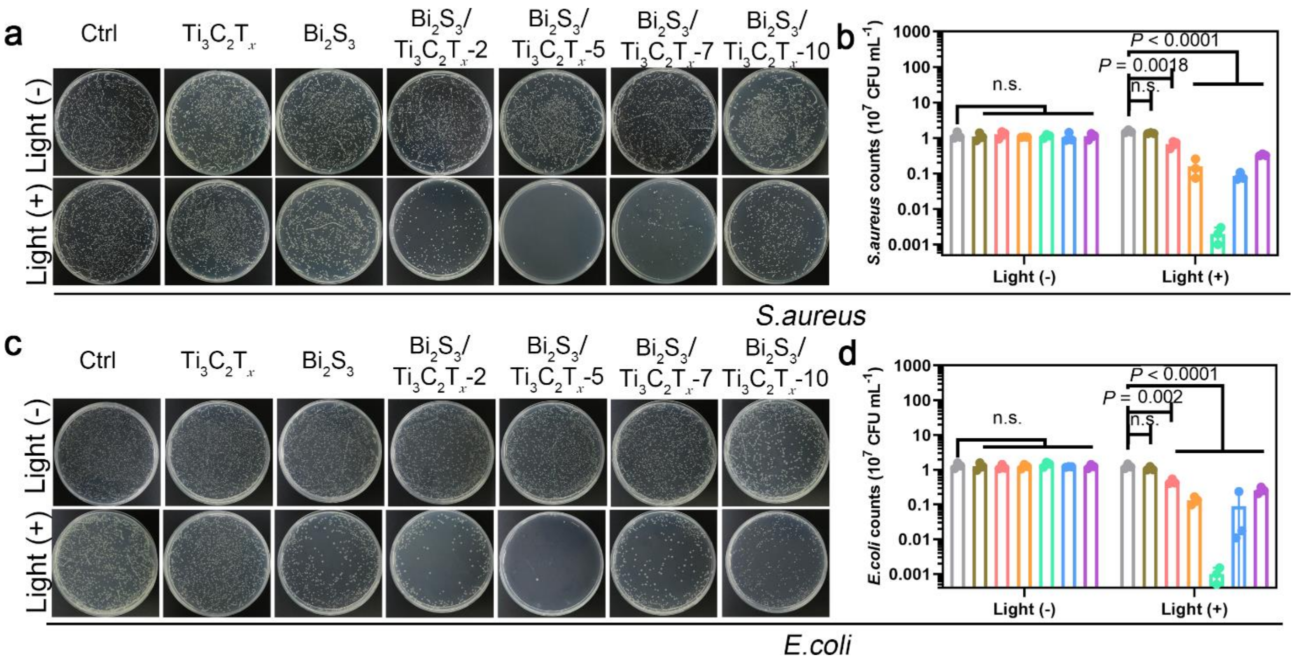

Supplementary Fig. 21 | Spread plate results and corresponding strain counts. a, b, Spread plate results (**a**) and corresponding strain counts (**b**) of *S. aureus*. **c, d,** Spread plate results (**c**) and corresponding strain counts (**d**) of *E. coli* treated with $Ti_3C_2T_x$, Bi_2S_3, $Bi_2S_3/Ti_3C_2T_x$-2, $Bi_2S_3/Ti_3C_2T_x$-5, $Bi_2S_3/Ti_3C_2T_x$-7, and $Bi_2S_3/Ti_3C_2T_x$-10 under 808 nm irradiation for 10 min after 24 h co-culturing. Data are presented as mean ± standard deviations from a representative experiment (n = 3 independent samples). P values were analysed by one-way ANOVA with Tukey's multiple comparisons post hoc test. Grey circles indicate the group of Ctrl, brown circles indicate the group of $Ti_3C_2T_x$, pink circles indicate the group of Bi_2S_3, orange circles indicate the group of $Bi_2S_3/Ti_3C_2T_x$-2, green circles indicate the group of $Bi_2S_3/Ti_3C_2T_x$-5, blue circles indicate the group $Bi_2S_3/Ti_3C_2T_x$-7, and purple circles indicate the group $Bi_2S_3/Ti_3C_2T_x$-10. Source data are provided as a Source Data file.

As shown in supplementary Fig. 21, the samples only exhibited negligible effects on bacterial survival in the dark. In contrast, under 808 nm NIR light irradiation for 10 min, the number of

bacterial colonies had significant difference among these samples with the same concentration of 200 ppm. $Ti_3C_2T_x$ exhibited almost no antibacterial activity for both *S. aureus* and *E. coli* due to its poor photothermal effects and little photocatalytic properties. Thanks to its mild NIR photothermal and photocatalytic performance, the pristine Bi_2S_3 showed weak antibacterial efficacy. In contrast, under 10 min NIR light irradiation, $Bi_2S_3/Ti_3C_2T_x$-5 group exhibited highly effective bacteria-killing efficiency of 99.86% and 99.92% against *S. aureus* and *E. coli*, respectively.

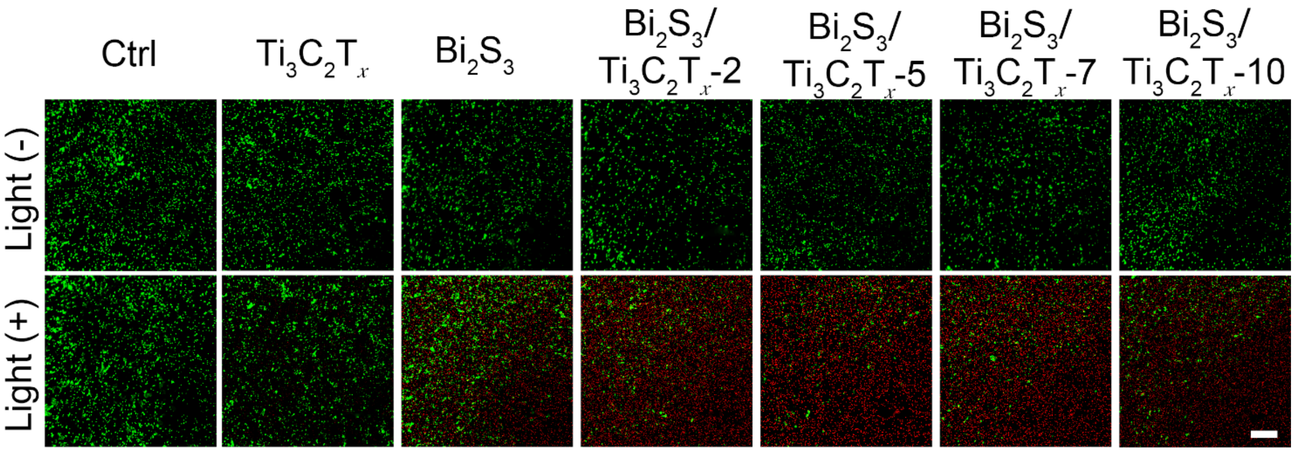

Supplementary Fig. 22 | Bacterial live/dead fluorescence staining of *S. aureus*. Bacterial live/dead fluorescence staining for $Ti_3C_2T_x$, Bi_2S_3, $Bi_2S_3/Ti_3C_2T_x$-2, $Bi_2S_3/Ti_3C_2T_x$-5, $Bi_2S_3/Ti_3C_2T_x$-7, and $Bi_2S_3/Ti_3C_2T_x$-10 in vitro (green fluorescence represents live bacteria, red fluorescence represents dead bacteria). The scale bar is 100 μm.

The results of qualitative bacterial viability were assessed by bacterial live/dead fluorescence staining in Supplementary Fig. 22. A great number of green fluorescence (live bacteria) was detected without NIR irradiation. In contrast, most of red fluorescence (dead bacteria) can be observed from Bi_2S_3 and $Bi_2S_3/Ti_3C_2T_x$-x groups. Obviously, only very few live bacteria can be observed for $Bi_2S_3/Ti_3C_2T_x$-5, suggesting the best antibacterial efficacy.

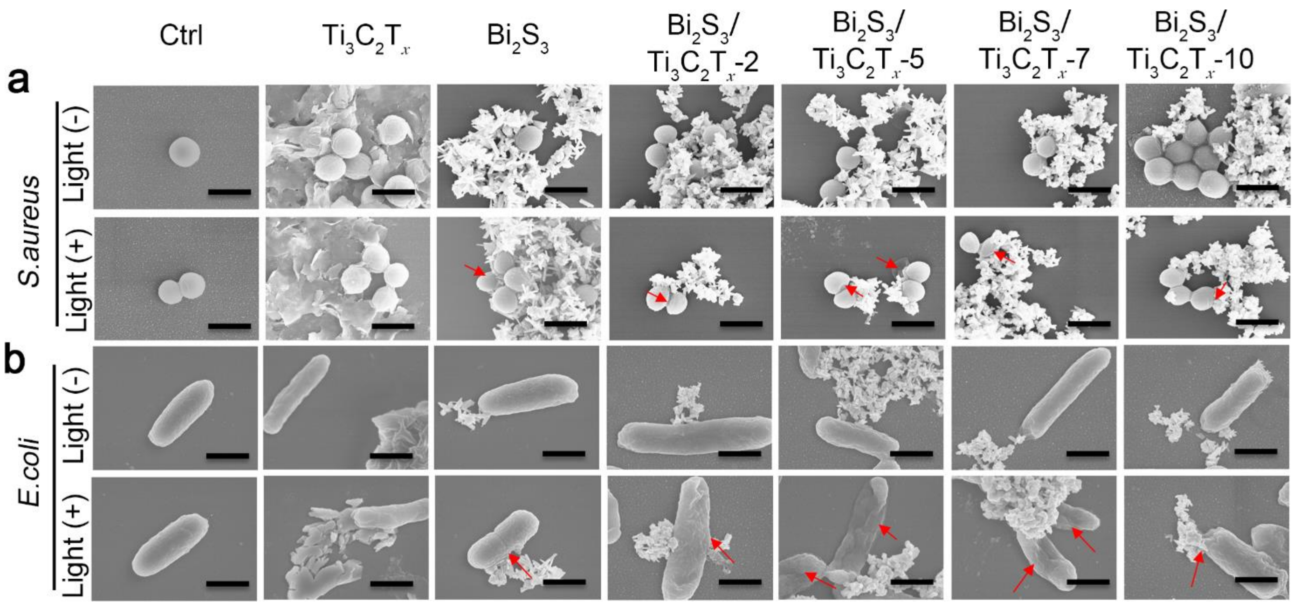

Supplementary Fig. 23 | The SEM antibacterial images. a, b, SEM antibacterial images for *S. aureus* (**a**) and *E. coli* (**b**) incubated with different samples of $Ti_3C_2T_x$, Bi_2S_3, $Bi_2S_3/Ti_3C_2T_x$-2, $Bi_2S_3/Ti_3C_2T_x$-5, $Bi_2S_3/Ti_3C_2T_x$-7, and $Bi_2S_3/Ti_3C_2T_x$-10. (The scale bar is 1 μm).

As shown in Supplementary Fig. 23, all as-prepared samples showed negligible antibacterial performance without light irradiation, evidenced by the normal spherical of *S. aureus* and regular rod of *E. coli*. Similarly, these smooth surface and regular shape also can be seen in $Ti_3C_2T_x$ group under NIR light. In contrast, the integrity of bacterial membrane in both Bi_2S_3 and $Bi_2S_3/Ti_3C_2T_x$-x groups was disrupted in different levels. The irreparable cellular membrane damage occurred in the bacteria of $Bi_2S_3/Ti_3C_2T_x$-5 group under 10 min light irradiation.

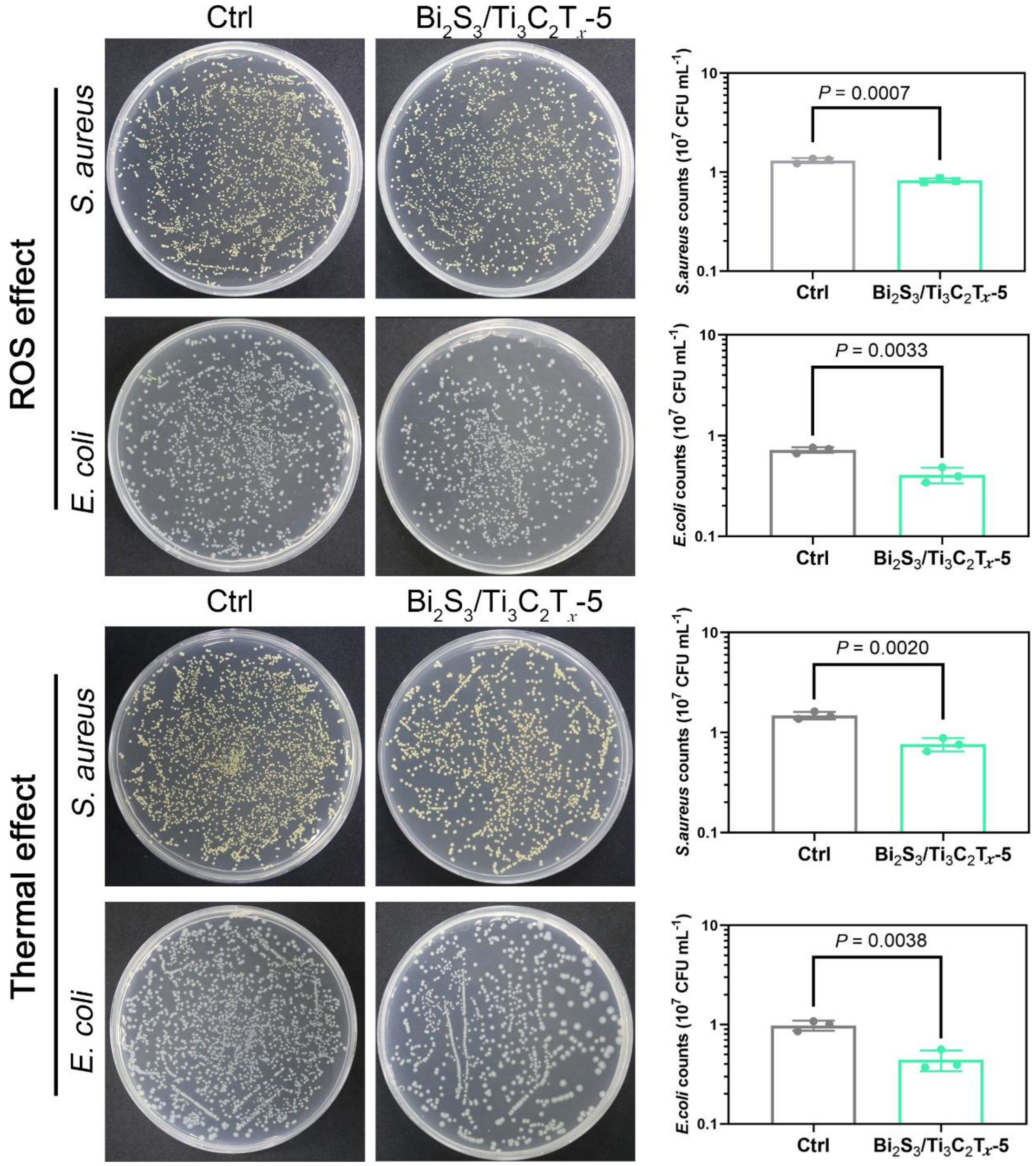

Supplementary Fig. 24 | The antibacterial performance for single ROS or photothermal effect alone. Spread plate results and corresponding strain counts of *S. aureus* and *E. coli*. Data are shown as mean ± standard deviations; $n = 3$ independent samples. *P* values were analysed by one-way ANOVA with Tukey's multiple comparisons post hoc test. Source data are provided as a Source Data file.

To verify the synergistic effect of ROS and photothermal effect, the antibacterial performance was assessed. Under single ROS effect, the antibacterial rate against *S. aureus* and *E. coli* was 37.06%

and 43.47%, respectively. In addition, under photothermal effect alone, the antibacterial efficiency of 48.51% against *S. aureus* and 54.88% against *E. coli* can be observed. The sum of the individual photothermal and photodynamic antibacterial effects (85.57% for *S. aureus* and 98.35% for *E. coli*) is less than the synergistic antibacterial effect (99.86% for *S. aureus* and 99.92% for *E. coli*). Thus, the synergy of ROS and photothermal effect can achieve much better antibacterial performance than single ROS or photothermal effect alone.

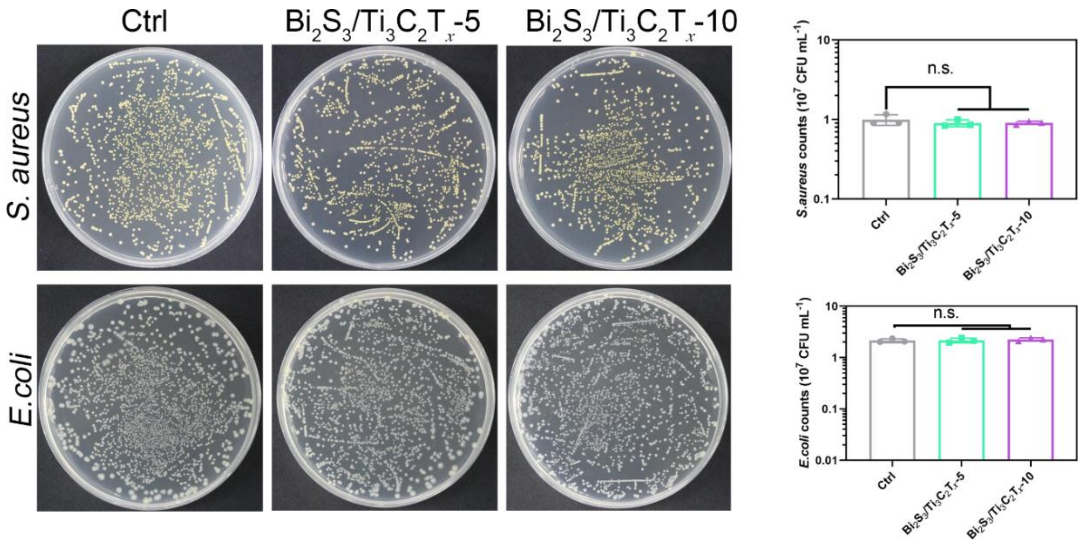

Supplementary Fig. 25 | The antibacterial performance in the dark. Spread plate results and corresponding strain counts of *S. aureus* and *E. coli* for 12 h in the dark. Data are shown as mean ± standard deviations; $n = 3$ independent samples. P values were analysed by one-way ANOVA with Tukey's multiple comparisons post hoc test. Source data are provided as a Source Data file.

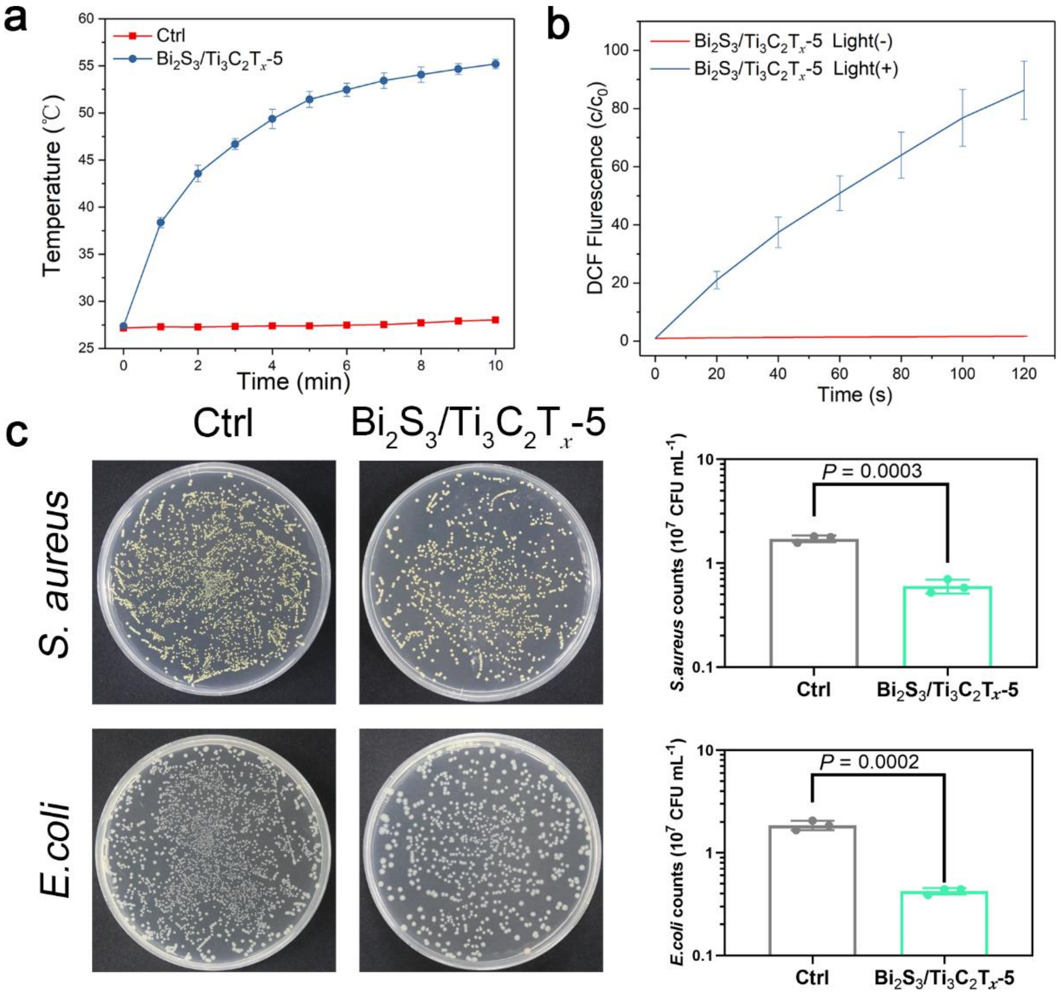

Supplementary Fig. 26 | The antibacterial performance of $Bi_2S_3/Ti_3C_2T_x$-5 under 660 nm (0.7 W cm^{-1}). a, Photothermal curves under 10 min light. **b,** ROS production with DCFH fluorescence probe (200 ppm). **c,** Spread plate results and corresponding strain counts of *S. aureus* and *E. coli*. Data are shown as mean ± standard deviations; n = 3 independent samples. P values were analysed by one-way ANOVA with Tukey's multiple comparisons post hoc test. Source data are provided as a Source Data file.

To verify the antibacterial performance of the samples under 660 nm light irradiation, photothermal curves and the yield of ROS were measured. As shown in Supplementary Fig. 26, the temperature

only reached to 55.2 ^{o}C within 10 min and the yield of ROS was much lower than that of $Bi_2S_3/Ti_3C_2T_x$-5 under 808 nm light irradiation. The antibacterial efficacy only reached to 65.08% and 77.17% against *S. aureus* and *E. coli*, which is lower than that under 808 nm light irradiation. This is attributed to the low temperature and ROS yield under 660 nm light.

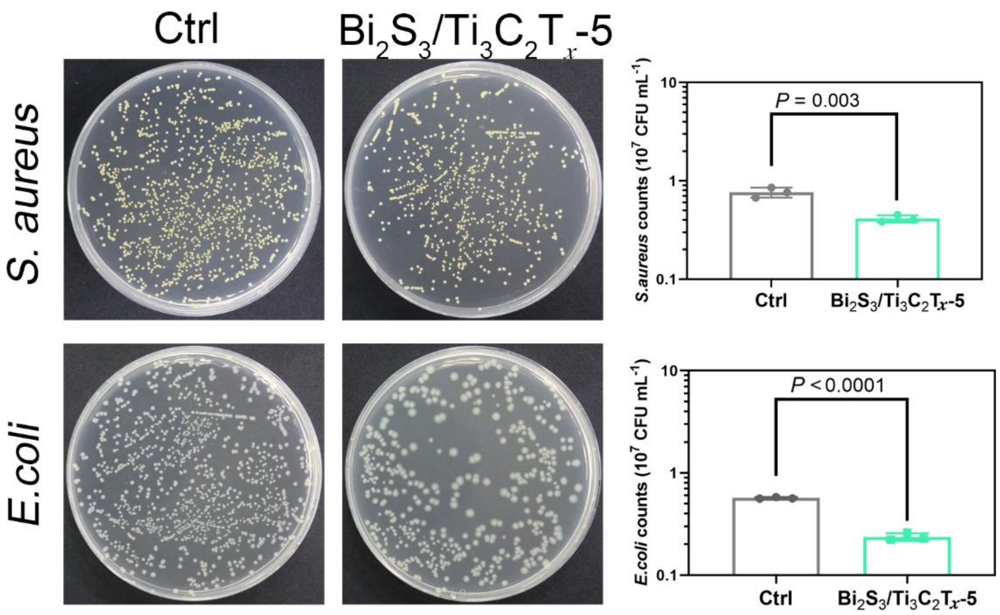

Supplementary Fig. 27 | The antibacterial performance of $Bi_2S_3/Ti_3C_2T_x$-5 under solar irradiation (0.2 W cm^{-2}). Spread plate results and corresponding strain counts of *S. aureus* and *E. coli*. Data are shown as mean ± standard deviations; $n = 3$ independent samples. P values were analysed by one-way ANOVA with Tukey's multiple comparisons post hoc test. Source data are provided as a Source Data file.

The antibacterial activity of $Bi_2S_3/Ti_3C_2T_x$-5 under simulated solar irradiation was also assessed. As shown in Supplementary Fig. 27, $Bi_2S_3/Ti_3C_2T_x$-5 exhibited poor antibacterial performance. The antibacterial efficacy was only 45.95% and 58.34% against *S. aureus* and *E. coli*, respectively, which is much lower than that under 808 nm light irradiation.

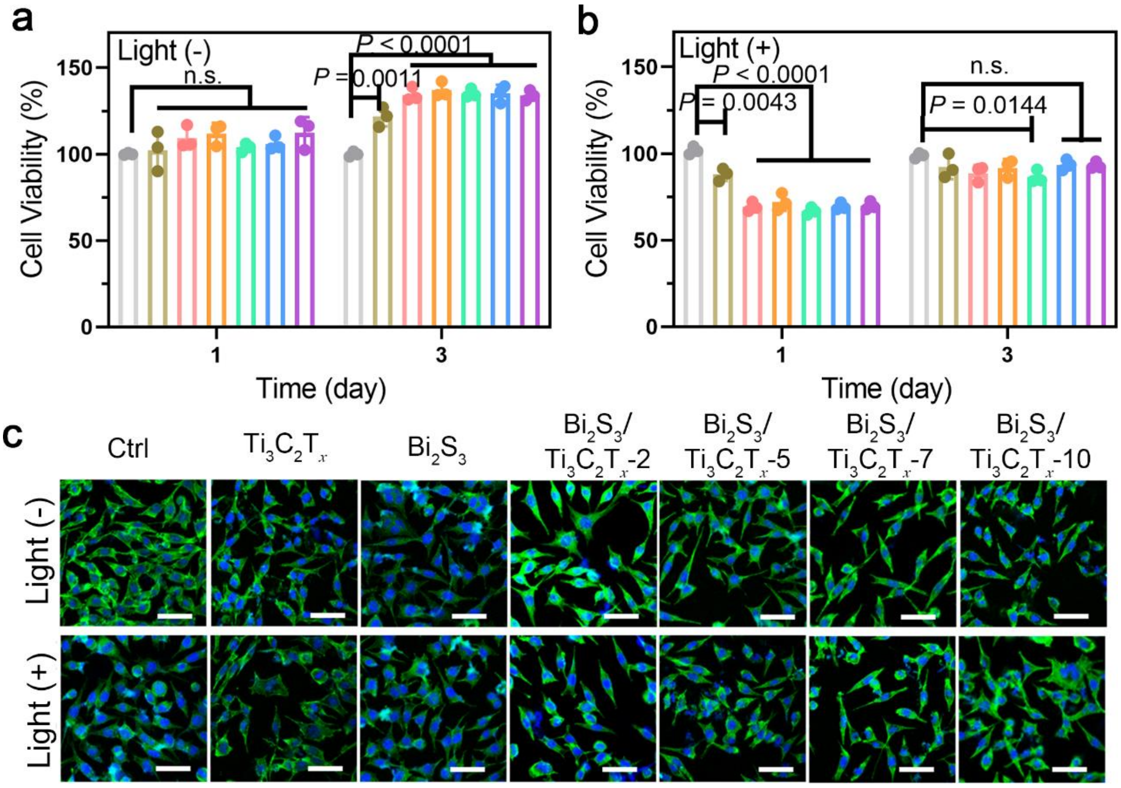

Supplementary Fig. 28 | The cell biocompatibility in vitro of materials. a, Cell viability for 1 and 3 d in the dark. **b,** Cell viability for 1 and 3 d with 808 nm light for 10 min. **c,** Cell fluorescence staining of control, $Ti_3C_2T_x$, Bi_2S_3, $Bi_2S_3/Ti_3C_2T_x$-2, $Bi_2S_3/Ti_3C_2T_x$-5, $Bi_2S_3/Ti_3C_2T_x$-7 and $Bi_2S_3/Ti_3C_2T_x$-10. The scale bars are 50 μm. Data are presented as mean ± standard deviations from a representative experiment (n = 3 independent samples). *P* values were analysed by one-way ANOVA with Tukey's multiple comparisons post hoc test. Grey circles indicate the group of Ctrl, brown circles indicate the group of $Ti_3C_2T_x$, pink circles indicate the group of Bi_2S_3, orange circles indicate the group of $Bi_2S_3/Ti_3C_2T_x$-2, green circles indicate the group of $Bi_2S_3/Ti_3C_2T_x$-5, blue circles indicate the group $Bi_2S_3/Ti_3C_2T_x$-7, and purple circles indicate the group $Bi_2S_3/Ti_3C_2T_x$-10. Source data are provided as a Source Data file.

As far as a good biomaterial was concerned, it was imperative to consider cell viability and cell growth. As is reported, $Ti_3C_2T_x$ MXene was considered to be non-toxic to living tissues and

biocompatible, in which the element of C was necessary for the structure of biological tissue and Ti (a transition metal element) was an inert element for organisms[20]. Meanwhile, Bi was considered to be low toxicity and good tolerance even at high concentration. What was more, Bi_2S_3 only had been proven to possess excellent biocompatibility and did not leave residue in the organisms[21]. Although these elements, including Ti, C, Bi and S were non-toxic and cytocompatibility, the structure and morphology also had great effect on cell viability, so it was necessary to investigate cell viability. As shown in Supplementary Fig. 28a and 28b, the qualitative results of cell viability after incubation with $Ti_3C_2T_x$, Bi_2S_3, $Bi_2S_3/Ti_3C_2T_x$-2, $Bi_2S_3/Ti_3C_2T_x$-5, $Bi_2S_3/Ti_3C_2T_x$-7, and $Bi_2S_3/Ti_3C_2T_x$-10 for 1 and 3 d were measured by methyl thiazolyl tetrazolium (MTT) assay. The OD values corresponding to the cell numbers at 490 nm were tested. Whether or not it was irradiated, the number of cells increased with the extension of incubation time. Without 808 NIR irradiation, there was no significant difference among different groups after 1 d, whereas an increased trend occurred compared to that of control group after 3 d cultivation, especially for $Bi_2S_3/Ti_3C_2T_x$-x and Bi_2S_3 groups, indicating the introduce of Bi_2S_3 had no appreciable cytotoxicity and the cell viability was not suppressed. When exposed under NIR light, the number of cells for $Ti_3C_2T_x$, Bi_2S_3 and $Bi_2S_3/Ti_3C_2T_x$-x decreased after 1 d incubation, indicating the certain side effect of photothermal and photodynamic effects on cells, decreasing of cell proliferation rate. However, cells can quickly recover for short time, evidenced by the higher cell viability in 3 d.

To further illustrate the cell viability, the spreading activity of NIH-3T3 cells was qualitatively analyzed by fluorescence staining. The cytoskeleton of cells co-cultured with different samples (200 ppm) were observed by laser scanning confocal microscope (CLSM) to visualize the F-actin and cell nuclei, respectively (Supplementary Fig. 28c). Whether or not it was illuminated, spindle structure with clear cytoskeleton and obvious filopodia extensions can be observed after 24 h culturing. No prominent difference of cell morphology among all groups can be detected. This

result suggested short exposure time had no effect on cell morphology owing to the good compatibility of materials.

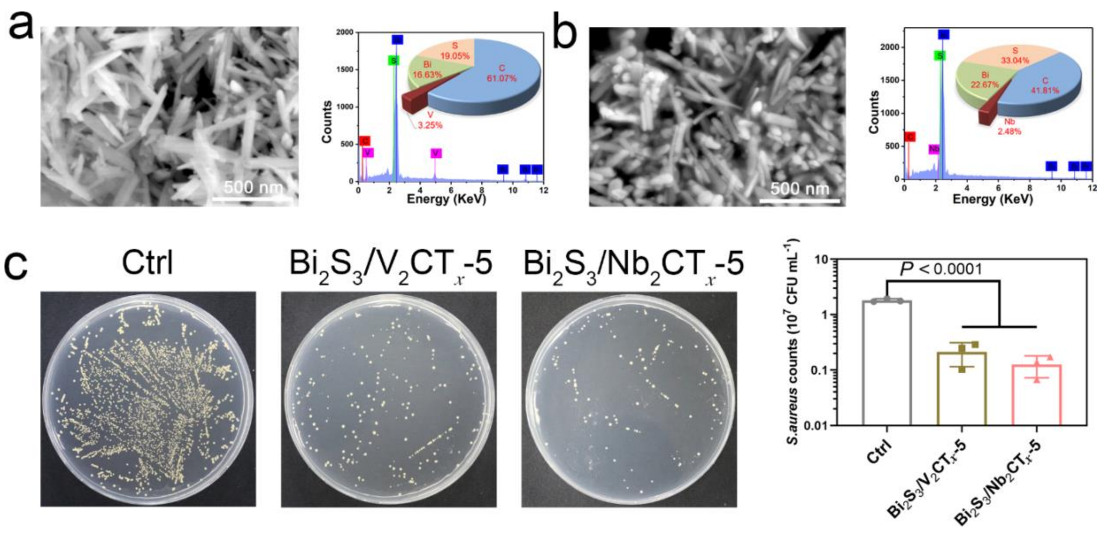

Supplementary Fig. 29 | The characterization and antibacterial properties of Bi_2S_3/V_2CT_x-5 and Bi_2S_3/Nb_2CT_x-5. a, SEM images and corresponding EDS analysis of Bi_2S_3/V_2CT_x-5. **b,** SEM images and corresponding EDS analysis of Bi_2S_3/Nb_2CT_x-5. **c,** The spread plate results and corresponding strain counts of *S. aureus* under 808 nm light irradiation (0.7 W cm^{-2}). Data are shown as mean ± standard deviations; $n = 3$ independent samples. P values were analysed by one-way ANOVA with Tukey's multiple comparisons post hoc test. Source data are provided as a Source Data file.

To verify that the Bi_2S_3 can act as a versatile antibacterial material on different MXene, V_2CT_x and Nb_2CT_x were chosen as composite materials due to their good biocompatibiity[22]. The antibacterial activity of Bi_2S_3/V_2CT_x-5 and Bi_2S_3/Nb_2CT_x-5 was assessed by the same spread plate method. As it can be seen in Supplementary Fig. 29a and 29b, the obvious nanorods structure can be seen. And the length of Bi_2S_3/V_2CT_x-5 and Bi_2S_3/Nb_2CT_x-5 is slightly longer than $Bi_2S_3/Ti_3C_2T_x$-5. The antibacterial performance under 808 nm light irradiation was exhibited in Supplementary Fig. 29c. The antibacterial efficiency of Bi_2S_3/V_2CT_x-5 and Bi_2S_3/Nb_2CT_x-5 can reach to 88.23 ± 4.39%, 93.03± 2.43%, respectively. Thus, Bi_2S_3/V_2CT_x-5 and Bi_2S_3/Nb_2CT_x-5 also exhibited excellent antibacterial performance.

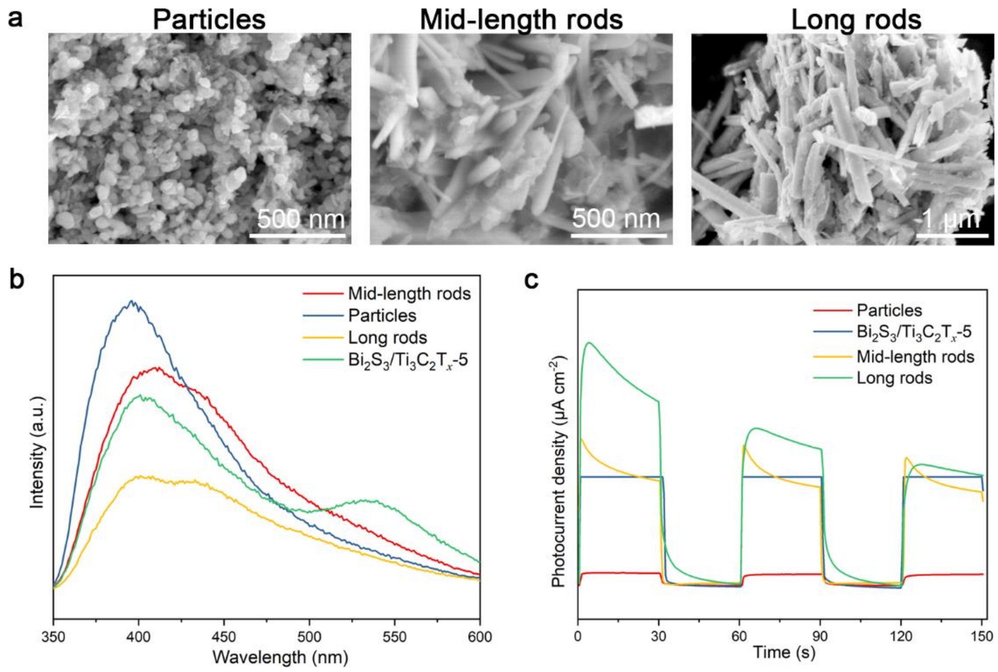

Supplementary Fig. 30 | The characterization and photocatalytic properties of $Bi_2S_3/Ti_3C_2T_x$-5 with different rods. a, SEM images **b,** PL spectra tested with 325 nm excitation wavelength from 350 nm to 600 nm. **c,** Photocurrent response under 808 nm NIR irradiation. Source data are provided as a Source Data file.

To demonstrate the effect of Bi_2S_3 rod lengths on the rate of photogenerated electrons, the different lengths of Bi_2S_3 was prepared. As shown in Supplementary Fig. 30a, the different lengths can be observed from particles to long rods (more than 1 μm). PL spectra can reflect the separation efficiency of photogenerated electrons and holes. The longer rods showed a lower photogenerated electron–hole recombination rate, indicating the efficient transfer and separation of photoexcited electron–hole. Similarly, there was an increasing tendency in photocurrent with the increase of length. PL spectra and photocurrent density showed that the length of Bi_2S_3 had an influence on the separation of electron-hole pairs. This similar result was also demonstrated by TiO_2 nanowires length[23].

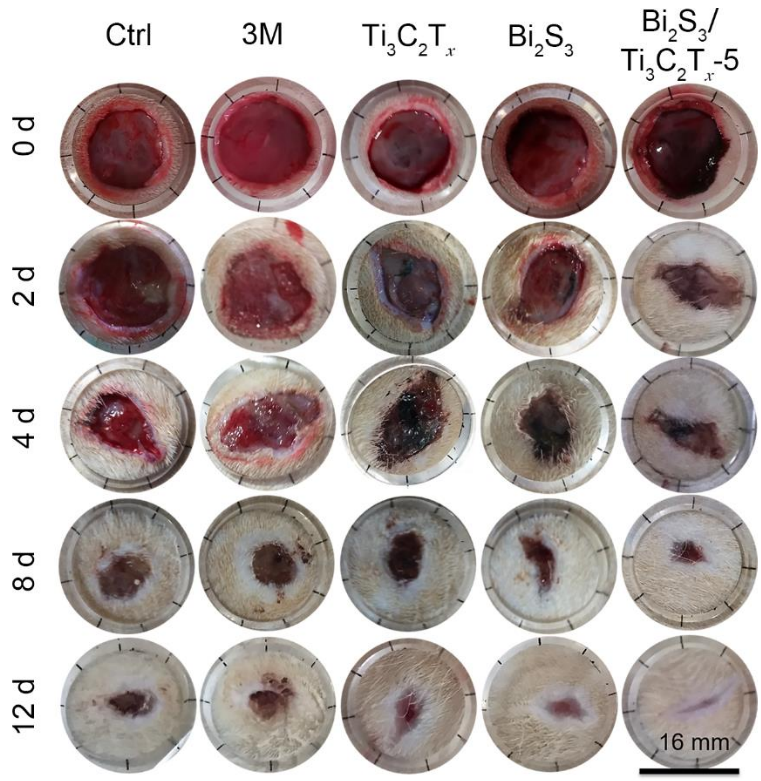

Supplementary Fig. 31 | The infected wound photographs. Representative infected wound photographs of rats collected on 0, 2, 4, 8, and 12 d for control, 3M wound dressing, $Ti_3C_2T_x$, Bi_2S_3, and $Bi_2S_3/Ti_3C_2T_x$-5.

The wound healing pictures can be seen in Supplementary Fig. 31, at 2 and 4 d after bacterial infection, obvious suppuration and inflammatory response was observed in the groups of the control, 3M, $Ti_3C_2T_x$, and Bi_2S_3. As a contrast, the wounds treated by $Bi_2S_3/Ti_3C_2T_x$-5 almost had no infection and even the wound areas had started to decrease. In consistent with the in vitro antibacterial activity, $Bi_2S_3/Ti_3C_2T_x$-5 also showed excellent antibacterial performance in vivo. After 12 d, the wounds of $Bi_2S_3/Ti_3C_2T_x$-5 group had almost healed up, which showed significantly faster healing rate than other groups, where large wounds still be visualized. Remarkably, combinational photothermal and ROS synergistic treatment displayed the best wound healing effects.

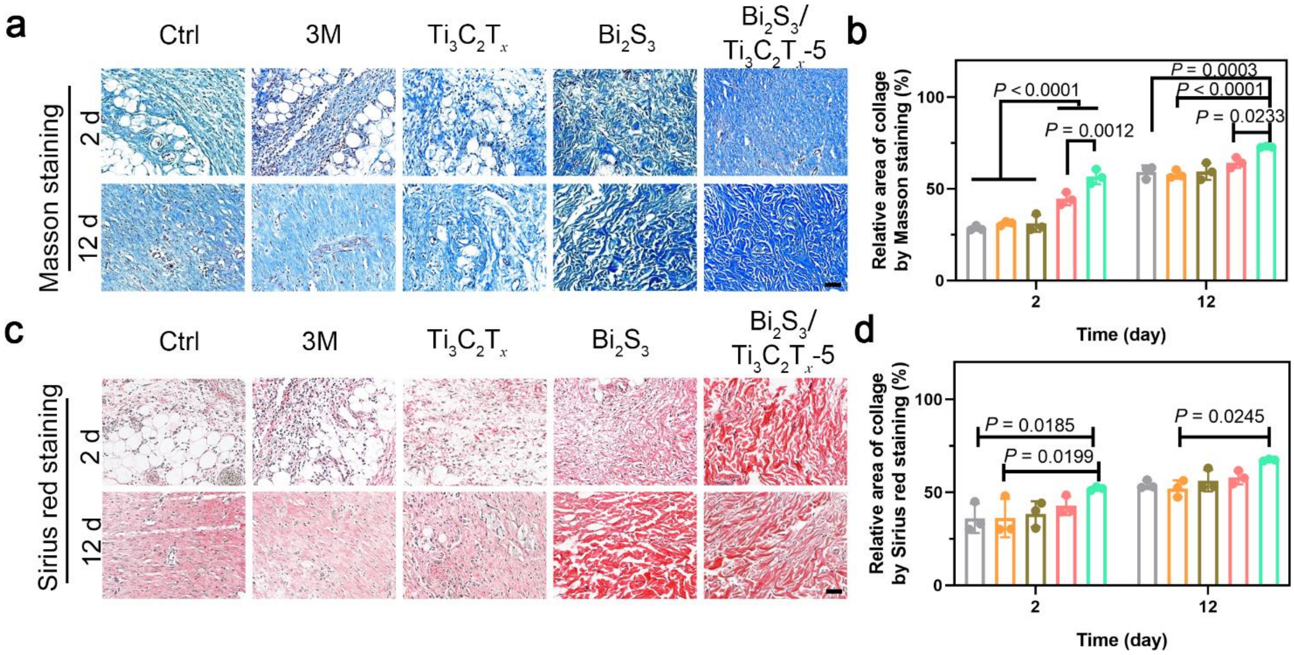

Supplementary Fig. 32 | Masson staining and Sirius red staining images. a, b, Masson staining (**a**) and the corresponding quantitative result of collagen (**b**). **c, d,** Sirius red staining (**c**) and the corresponding quantitative result of collagen (**d**) on 2, 12 d for control, 3M wound dressing, $Ti_3C_2T_x$, Bi_2S_3, and Bi_2S_3/$Ti_3C_2T_x$-5 groups. The scale bars are 50 µm. Data are shown as mean ± standard deviations; *n* = 3 independent samples. *P* values were analysed by one-way ANOVA with Tukey's multiple comparisons post hoc test. Source data are provided as a Source Data file.

Masson staining and Sirius red staining were used to assess the formation of collagen fiber in Supplementary Fig. 32. After 2 d treatment, different level of tissue necrosis was observed in the control, 3M, and $Ti_3C_2T_x$ groups. The collagen fibers in Bi_2S_3 group also showed a bit loose and disordered structure compared to that in Bi_2S_3/$Ti_3C_2T_x$-5 group, which had widespread collagen deposition and dense collagen fibers. As the wounds gradually healed, the deposition of collagen fibers in each group gradually increased. Abundant collagen fibers and newly formed blood vessels had been seen around the wounds, especially for Bi_2S_3/$Ti_3C_2T_x$-5. The dense and organized collagen

fibers were deposited, suggesting the excellent biological performance of $Bi_2S_3/Ti_3C_2T_x$-5 during the process of wound healing. The amount of collagen deposition in the wounds was the relatively quantitatively determined. The collagen coverage rate of $Bi_2S_3/Ti_3C_2T_x$ -5 was about 56.62% and 71.57% for Masson staining and 52.07% and 66.39% for Sirius red staining, respectively, which was significantly higher than those of the control groups.

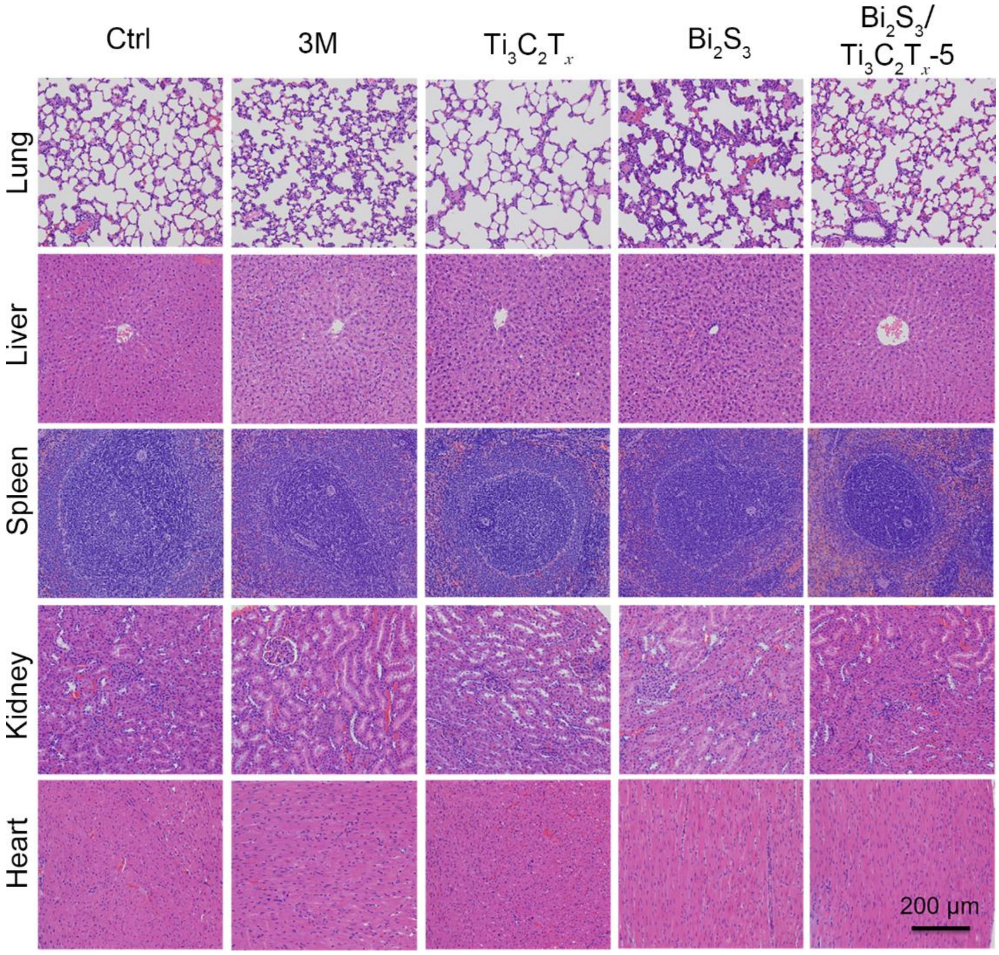

Supplementary Fig. 33 | H&E staining of heart, liver, spleen, lung, and kidney treated with control, 3M wound dressing, $Ti_3C_2T_x$, Bi_2S_3, and $Bi_2S_3/Ti_3C_2T_x$-5. The scale bars are 200 μm.

The in vivo biosafety was evaluated by H&E staining of major rat organs shown in Supplementary Fig. 33. No obvious toxicology can be visualized on heart, liver, spleen, lung, and kidney, indicating the excellent in vivo biosafety of all groups including $Ti_3C_2T_x$, Bi_2S_3 and $Bi_2S_3/Ti_3C_2T_x$-5.

Supplementary Table 1. Electrochemical parameters derived from EIS fitting for different samples

Samples	R_s (Ω cm^2)	CPE (μF cm^{-2})	n	Z_w (Ω cm^2)
Bi_2S_3 Dark	27.3	8.56	0.99	211.2
Bi_2S_3 Light	33.01	5.19	0.99	155.1
$Bi_2S_3/Ti_3C_2T_x$-2 Dark	37.4	7.18	0.96	88.27
$Bi_2S_3/Ti_3C_2T_x$-2 Light	38.65	7.06	0.98	85.59
$Bi_2S_3/Ti_3C_2T_x$-5 Dark	50.67	6.06	0.96	39.5
$Bi_2S_3/Ti_3C_2T_x$-5 Light	49.05	5.05	0.96	32.78
$Bi_2S_3/Ti_3C_2T_x$-7 Dark	33.57	7.01	0.97	43.95
$Bi_2S_3/Ti_3C_2T_x$-7 Light	33.38	7.05	0.97	39.65
$Bi_2S_3/Ti_3C_2T_x$-10 Dark	34.93	8.33	0.99	79.92
$Bi_2S_3/Ti_3C_2T_x$-10 Light	36.44	5.81	0.98	53.55

Supplementary Table 2 The comparison between $Bi_2S_3/Ti_3C_2T_x$ and Ti_3C_2/Ag

	$Bi_2S_3/Ti_3C_2T_x$	Ti_3C_2/Ag membranes
Materials	Bi_2S_3, 5wt% $Ti_3C_2T_x$	21% Ag, Ti_3C_2
Concentration	200 ppm	-
Condition	10 min, 808 nm irradiation	24 h at 36 ℃
Bacterial concentration ($CFU\ mL^{-1}$)	10^7	$2*10^3$
Antibacterial rate	99.86% of *S. aureus*, 99.92% of *E. coli*	more than 99% *E. coli*
Antibacterial mechanism	The synergistic effect of Photodynamic and photothermal role	The release of Ag, contact with bacteria
Biocompatibility	Good	-

Supplementary Methods

Synthesis of $Bi_2S_3/Ti_3C_2T_x$ particles. To prepared $Bi_2S_3/Ti_3C_2T_x$ particles, $Ti_3C_2T_x$ aqueous solution (pH=6.42) was sonicated by ultrasonic cell grinder. And then 291 mg of $Bi(NO_3)_3·5H_2O$ was dissolved in deionized water containing a 7.5 mL of $Ti_3C_2T_x$ aqueous solution and stirred for 2 h. Subsequently, 81 mg of thioacetamide (C_2H_5NS) was added and stirred for 30 min. The mixed solution was transferred into a 50 mL Teflon-lined autoclave to maintain 160 °C for 8 h. After cooling down, the final black precipitate was rinsed and centrifuged with deionized water, and dried in a vacuum drying oven.

Synthesis of $Bi_2S_3/Ti_3C_2T_x$ mid-long rods. To prepared $Bi_2S_3/Ti_3C_2T_x$ mid-long rods, $Ti_3C_2T_x$ aqueous solution (pH=6.42) was sonicated by ultrasonic cell grinder. And then 97 mg of $Bi(NO_3)_3·5H_2O$ was dissolved in deionized water containing a 2.5 mL of $Ti_3C_2T_x$ aqueous solution and stirred for 2 h. Subsequently, 27 mg of thiourea was added and stirred for 30 min. The mixed solution was transferred into a 50 mL Teflon-lined autoclave to maintain 180 °C for 12 h. After cooling down, the final black precipitate was rinsed and centrifuged with deionized water, and dried in a vacuum drying oven.

Synthesis of $Bi_2S_3/Ti_3C_2T_x$ long rods. To prepared $Bi_2S_3/Ti_3C_2T_x$ long rods, $Ti_3C_2T_x$ aqueous solution (pH=6.42) was sonicated by ultrasonic cell grinder. And then 0.73 g of $Bi(NO_3)_3·5H_2O$ was dissolved in 19 mL of $Ti_3C_2T_x$ aqueous solution and stirred for 2 h. 6.25 mmol L^{-1} thioacetamide solution was dipped in the above solution. And then 0.76 g carbamide was added to this solution. Transfer into a 50 mL Teflon-lined autoclave to maintain 120 °C for 12 h. After cooling down, the final black precipitate was rinsed and centrifuged with deionized water, and dried in a vacuum drying oven.

Supplementary References

1. Fan, B. et al. Investigation of adjacent spacing dependent microwave absorption properties of lamellar structural $Ti_3C_2T_x$ MXenes. Advanced Powder Technology. **31,** 808-815 (2020).
2. Nie, G., Lu, X., Lei, J., Yang, L. & Wang, C.J.E.A. Facile and controlled synthesis of bismuth sulfide nanorods-reduced graphene oxide composites with enhanced supercapacitor performance. *Electrochim. Acta* **154,** 24-30 (2015).
3. Ren, C.E. et al. Porous two-dimensional transition metal carbide (MXene) flakes for high-performance Li-ion storage. *Chem. Electro. Chem.* **3,** 689-693 (2016).
4. Ni, J. et al. Strongly coupled Bi_2S_3@CNT hybrids for robust lithium storage. *Adv. Energy Mater.* **4,** 1400798 (2014).
5. Liu, Z. et al. Large-scale synthesis of ultralong Bi_2S_3 nanoribbons via a solvothermal process. *Adv. Mater.* **15,** 936-940 (2003).
6. Liao, Y. et al. 2D-layered Ti_3C_2 MXenes for promoted synthesis of NH3 on P25 photocatalysts. *Applied Catalysis B: Environmental* **273** (2020).
7. Ghidiu, M., Lukatskaya, M.R., Zhao, M.Q., Gogotsi, Y. & Barsoum, M.W. Conductive two-dimensional titanium carbide 'clay' with high volumetric capacitance. *Nature* **516**, 78-81 (2014).
8. Cao, S., Shen, B., Tong, T., Fu, J. & Yu, J. 2D/2D heterojunction of ultrathin MXene/Bi_2WO_6 nanosheets for improved photocatalytic CO_2 reduction. *Adv. Funct. Mater.* **28,** 1800136 (2018).
9. Shamsabadi, A.A. et al. Pushing rubbery polymer membranes to be economic for CO_2 separation: embedment with $Ti_3C_2T_x$ MXene nanosheets. *ACS Appl. Mater. Interfaces* **12,** 3984-3992 (2020).
10. Xiao, Y., Cao, H., Liu, K., Zhang, S. & Chernow, V. The synthesis of superhydrophobic Bi_2S_3

complex nanostructures. *Nanotechnology* **21**, 145601 (2010).

11. Ma, Z., Zhou, X., Deng, W., Lei, D. & Liu, Z. 3D porous MXene (Ti_3C_2)/reduced graphene oxide hybrid films for advanced lithium storage. *ACS Appl. Mater. Interfaces* **10,** 3634-3643 (2018).
12. Halim, J. et al. Transparent conductive two-dimensional titanium Carbide epitaxial thin films. *Chem. Mater.* **26**, 2374-2381 (2014).
13. Li, Y. et al. A general Lewis acidic etching route for preparing MXenes with enhanced electrochemical performance in non-aqueous electrolyte. *Nat. Mater.* **19**, 894-899 (2020).
14. Geioushy, R. A., El-Sheikh, S. M., Azzam, A. B., Salah, B. A. & El-Dars, F. M. One-pot fabrication of $BiPO_4/Bi_2S_3$ hybrid structures for visible-light driven reduction of hazardous Cr(VI). *J Hazard Mater.* **381,** 120955 (2020).
15. Li, C. et al. Local charge distribution engineered by Schottky heterojunctions toward urea electrolysis. *Adv. Energy Mater.* **8,** 1801775 (2018).
16. Zhao, R., Di, L. N., Zhao, X. Z., Wang, C. & Zhang, G. A. Measuring surface temperature and grading pathological changes of airway tissue in a canine model of inhalational thermal injury. *Burns* **39,** 767-775 (2013).
17. Bernechea, M., Cao,Y. & Konstantatos, G. Size and bandgap tunability in Bi_2S_3 colloidal nanocrystals and its effect in solution processed solar cells. *J. Mater. Chem. A* **3,** 20642-20648 (2015).
18. Wang, H. et al. Electrical promotion of spatially photoinduced charge separation via interfacial-built-in quasi-alloying effect in hierarchical $Zn_2In_2S_5/Ti_3C_2(O, OH)_x$ hybrids toward efficient photocatalytic hydrogen evolution and environmental remediation. *Appl. Catal. B* **245,** 290-301 (2019).
19. He, R. et al. First-principles investigation of native point defects in two-dimensional Ti_3C_2. *Comput. Theor. Chem.* **1150,** 26-39 (2019).

20. Huang, K., Li, Z., Lin, J., Han, G. & Huang, P. Two-dimensional transition metal carbides and nitrides (MXenes) for biomedical applications. *Chem. Soc. Rev.* **47,** 5109-5124 (2018).

21. Liu, Y. et al. Fundamental theory of biodegradable metals-definition, criteria, and design. *Adv. Funct. Mater.* **29,** 1805402 (2019).

22. Zada, S. et al. Algae extraction controllable delamination of Vanadium Carbide nanosheets with enhanced near‐infrared photothermal performance. *Angew. Chem.* **132**, 6663-6668 (2020).

23. Hwang, Y. J., Hahn, C., Liu, B., & Yang, P. Photoelectrochemical properties of TiO_2 nanowire arrays: a study of the dependence on length and atomic layer deposition coating. *ACS Nano* **6**, 5060-5069, (2012).
